# Progesterone Receptor Membrane Component 1 suppresses the p53 and Wnt/β-catenin pathways to promote human pluripotent stem cell self-renewal

**DOI:** 10.1038/s41598-018-21322-z

**Published:** 2018-02-14

**Authors:** Ji Yea Kim, So Young Kim, Hong Seo Choi, Min Kyu Kim, Hyun Min Lee, Young-Joo Jang, Chun Jeih Ryu

**Affiliations:** 1 Department of Integrative Bioscience and Biotechnology, Institute of Anticancer Medicine Development, Sejong University, Seoul, Korea; 2 Department of Nanobiomedical Science, BK21 PLUS Global Research Center for Regenerative Medicine, Dankook University, Cheonan, Korea

## Abstract

Progesterone receptor membrane component 1 (PGRMC1) is a multifunctional heme-binding protein involved in various diseases, including cancers and Alzheimer’s disease. Previously, we generated two monoclonal antibodies (MAbs) 108-B6 and 4A68 against surface molecules on human pluripotent stem cells (hPSCs). Here we show that PGRMC1 is the target antigen of both MAbs, and is predominantly expressed on hPSCs and some cancer cells. PGRMC1 is rapidly downregulated during early differentiation of hPSCs. Although PGRMC1 knockdown leads to a spread-out morphology and impaired self-renewal in hPSCs, PGRMC1 knockdown hPSCs do not show apoptosis and autophagy. Instead, PGRMC1 knockdown leads to differentiation of hPSCs into multiple lineage cells without affecting the expression of pluripotency markers. PGRMC1 knockdown increases cyclin D1 expression and decreases Plk1 expression in hPSCs. PGRMC1 knockdown also induces p53 expression and stability, suggesting that PGRMC1 maintains hPSC self-renewal through suppression of p53-dependent pathway. Analysis of signaling molecules further reveals that PGRMC1 knockdown promotes inhibitory phosphorylation of GSK-3β and increased expression of Wnt3a and β-catenin, which leads to activation of Wnt/β-catenin signaling. The results suggest that PGRMC1 suppresses the p53 and Wnt/β-catenin pathways to promote self-renewal and inhibit early differentiation in hPSCs.

## Introduction

Progesterone receptor membrane component 1 (PGRMC1/Sigma-2 receptor) is a 25 kDa multifunctional protein with a heme-binding moiety^1^. It is overexpressed in multiple types of cancer, and represents an important biomarker of the proliferative status of cancers^2–4^. PGRMC1 binds to amyloid β oligomer to enhance its neuronal toxicity in Alzheimer’s disease^5,6^. PGRMC1 is associated with a large number of functions, including progesterone signaling, steroidogenesis, regulation of cytochrome P450, vesicle trafficking, mitotic spindle and cell cycle regulation, promotion of autophagy, angiogenesis, anchorage-independent growth, invasive growth, and hypoxic biology^1,7^. PGRMC1 was originally isolated from porcine liver microsomal membranes as a component of a membrane associated progesterone-binding activity^8^. PGRMC1 contains a short N-terminal extracellular or luminal domain, a single trans-membrane domain, and a much longer cytoplasm domain^9,10^. Several studies have suggested that PGRMC1 is localized at various subcellular locations, including endoplasmic reticulum, Golgi apparatus, inner acrosomal membrane, plasma membrane and nucleus^10–13^. It has been also reported that PGRMC1 is a cytochrome *b*_5_ related protein^14^, and regulates cell proliferation and apoptosis through interaction between its cytochrome *b*_5_ binding domain and other binding partners including epidermal growth factor receptor (EGFR)^15^, plasminogen activator inhibitor RNA-binding protein-1^16^ and P450 proteins^17,18^. The heme-mediated dimerization of adjacent PGRMC1 monomers leads PGRMC1 to interact with cytochromes P450 and EGFR, causing enhanced proliferation, anti-apoptosis, and chemoresistance of cancer cells^18^. A recent study suggests that PGRMC1 expresses in a variety of primary stem cells, and PGRMC1 expression may serve to monitor stem cell differentiation^19^. However, the function and mechanism of action of PGRMC1 in human pluripotent stem cells (hPSCs) have not been studied.

Previously, we generated a panel of murine monoclonal antibodies (MAbs) against the surface molecules on undifferentiated human embryonic stem cells (hESCs) by using a modified decoy immunization strategy^20^. In this study, we show that 108-B6 and 4A68, two of the MAbs, bind to surface molecules on various hPSCs, but not to them on differentiated primary cells. The expression of 108-B6 and 4A68 antigens is rapidly downregulated during early differentiation of hPSCs. Mass spectrometry and Western blotting confirm that 108-B6 and 4A68 recognize PGRMC1 on the surface of hPSCs. To investigate the role of PGRMC1 in hPSCs, PGRMC1 was knocked down in hPSCs by siRNA. The results reveal that PGRMC1 promotes hPSC self-renewal and inhibits differentiation in hPSCs without alterations in the expression of pluripotency markers. Analysis of signaling molecules further reveals how PGRMC1 regulates hPSC self-renewal and differentiation.

## Results

### 108-B6 and 4A68 antigen is predominantly expressed on the surface of hPSCs but not on the surface of differentiated cells

To investigate cell surface molecules on hPSCs, previously, we generated 70 MAbs against surface molecules on undifferentiated hPSCs using a decoy immunization strategy^20^. The present study investigated 108-B6 and 4A68, two of the 70 MAbs. Flow cytometric analysis showed that 108-B6 and 4A68 bound to hPSC lines H1, H9 and CHA-hES4 (Table 1). 108-B6 and 4A68 also bound to human embryonal carcinoma lines NT-2/D1 and NCCIT. In contrast, 108-B6 and 4A68 did not bind to mouse embryonic stem cell (mESC) (R1 and E14Tg2a.4) and mouse embryonic fibroblasts (MEFs). 108-B6 and 4A68 did not bind to human primary cells, such as peripheral blood mononuclear cells (PBMCs) and human dermal fibroblast cells (HDFs), either. 108-B6 and 4A68 antigen expression was negative for a panel of human cancer cells except for SH-SY5Y, NCI-H69 and Huh7 (Table 1). 108-B6 antigen expression was weakly positive in human glioblastoma cells (U87-MG), while 4A68 antigen expression was weakly positive in two human non-small cell lung carcinoma cells (NCI-H522 and A549) (Table 1). Taken together, the results suggest that both 108-B6 and 4A68 antigen are expressed on the surface of hPSCs and some cancer cells.

**Table 1:** Binding profiles of 108-B6 and 4A68.

Name	Cell origin	108-B6	4A68
H9	Human embryonic stem cell	++	++
H1	Human embryonic stem cell	+	+
CHA-hES4	Human embryonal stem cell	++	++
NT-2/D1	Human embryonal carcinoma	++	+
NCCIT	Human embryonal carcinoma	++	++
R1	Mouse embryonic stem cell	−	−
E14Tg2a.4	Mouse embryonic stem cell	−	−
MEF	Mouse embryonic fibroblast	−	−
PBMC	Peripheral blood monocytes	−	−
HDF	Human dermal fibroblast	−	−
ACHN	Human renal cell carcinoma, kidney	−	−
A172	Human glioblastoma, brain	−	−
A375	Human malignant melanoma, skin	−	−
SH-SY5Y	Human neuroblastoma, brain	+	+
NCI-H69	Human small cell lung carcinoma, lung	+	+
NCI-H522	Human nonsmall cell lung carcinoma, lung	−	+
A549	Human nonsmall cell lung carcinoma, lung	−	+
Huh7	Human hepatocellular carcinoma, liver	+	+
SK-HEP-1	Human hepatocellular carcinoma, liver	−	−
AGS	Human gastric adenocarcinoma, stomach	−	−
MKN28	Human gastric adenocarcinoma, stomach	−	−
MDA-MB435	Human ductal carcinoma, breast	−	−
MCF7	Human ductal adenocarcinoma, breast	−	−
U87-MG	Human glioblastoma, brain	+	−
Colo-205	Human adenocarcinoma, colon	−	−

++, strong binding; +, weakly binding; −, no binding.

**Figure 1:**
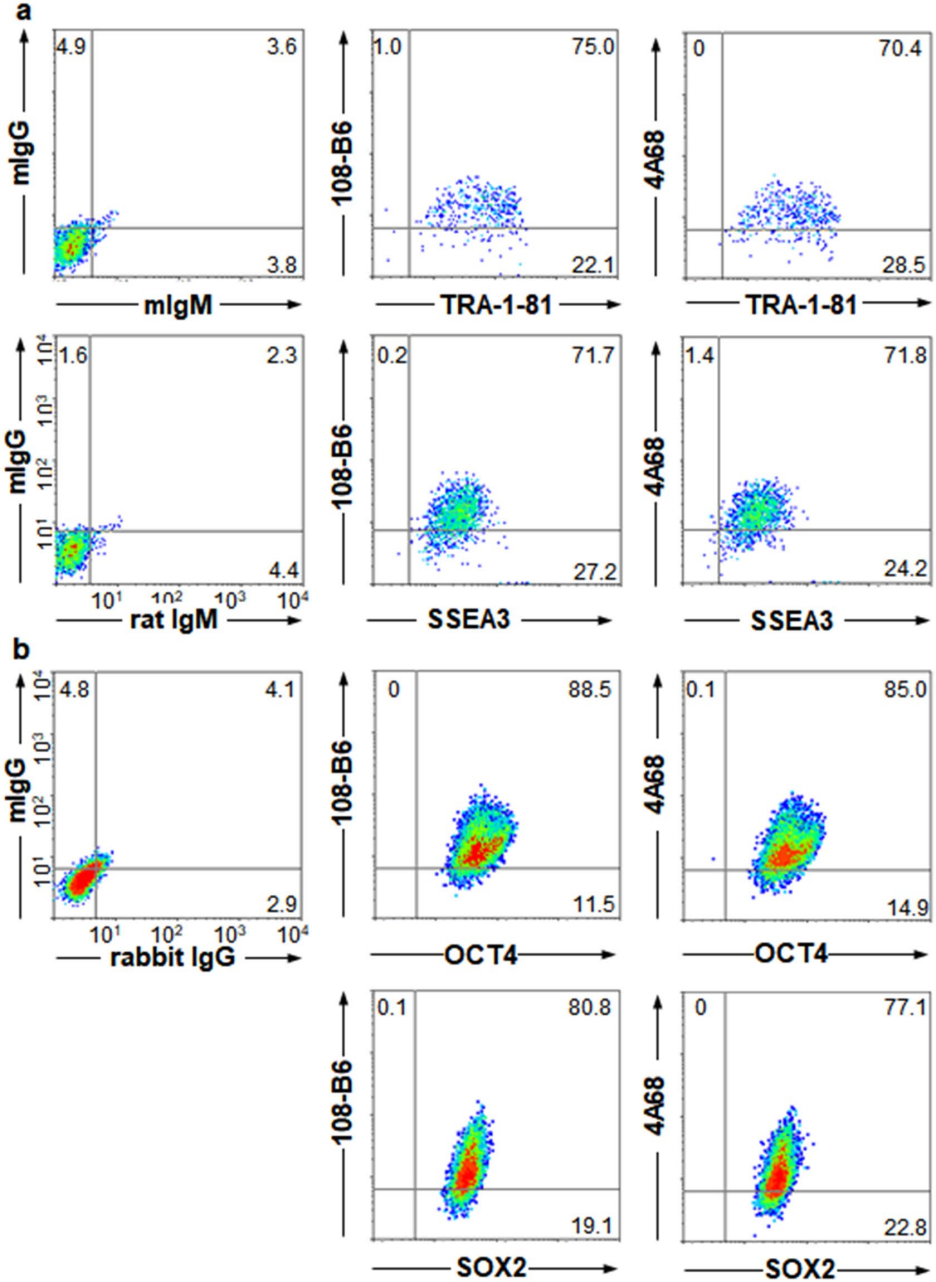
Expression of 108-B6 and 4A68 antigens is localized to undifferentiated and pluripotent hPSCs. (**a**) Multi-color flow cytometric analysis of hPSCs. H9 hPSCs were stained with MAbs (108-B6 or 4A68) and PE-conjugated anti-mouse IgG. The cells were then stained with anti-TRA-1-81 or anti-SSEA3 antibodies followed by incubation with FITC-conjugated anti-mouse IgM or anti-rat IgM, respectively, before analysis. (**b**) Multi-color intracellular flow cytometric analysis of hPSCs. H9 hPSCs were stained with MAbs 108-B6 or 4A68 and PE-conjugated anti-mouse IgG. The cells were fixed with 2% PFA and incubated in PBA containing 0.5% saponin. The cells were then stained with anti-OCT4 or anti-SOX2 antibodies followed by incubation with Alexa 488-cojugated anti-rabbit IgG before analysis.

We further examined 108-B6 and 4A68 specificity with H9 hPSCs using multicolor flow cytometric analysis. More than 71% of 108-B6-positive hPSCs were positive for the expression of TRA-1-81 and stage-specific embryonic antigen (SSEA-3) (Fig. 1a). Intracellular flow cytometry further showed that more than 80% of 108-B6-positive hPSCs were positive for the expression of octamer-binding transcription factor 4 (OCT4) and SRY (Sex Determining Region Y)-Box 2 (SOX2) (Fig. 1b). Similar results were obtained with 4A68 (Fig. 1a,b). Immunocytochemical analysis also showed that both 108-B6 and 4A68 antigens were colocalized with TRA-1-81 on H9 hPSCs (Supplementary Fig. 1a,b). The results suggest that 108-B6 and 4A68 antigens are predominantly expressed on the cell surface of pluripotent hPSCs but not on the surface of differentiated primary cells

### Cell surface-expressed PGRMC1 is the target antigen of both 108-B6 and 4A68

**Figure 2:**
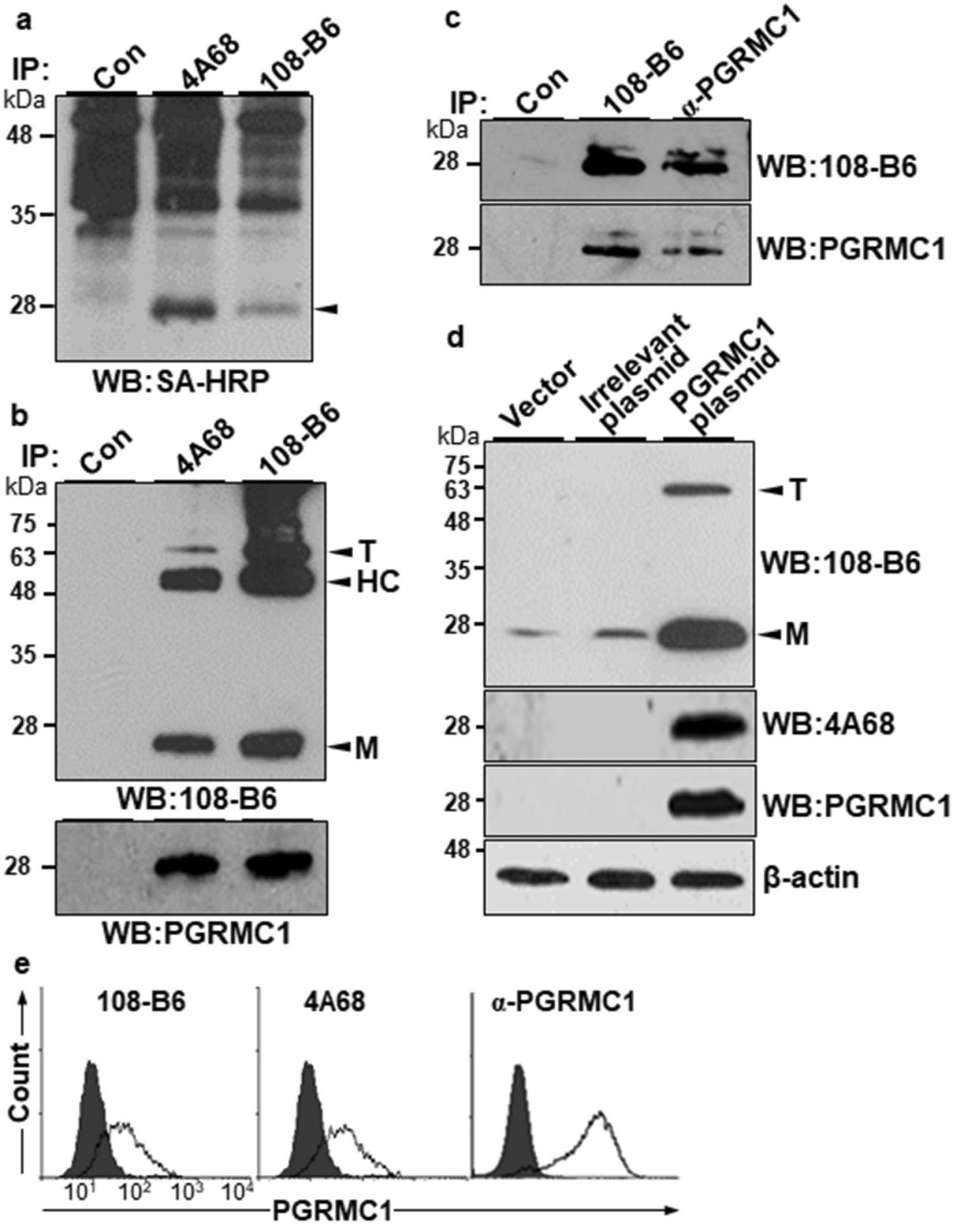
108-B6 and 4A68 recognize PGRMC1 on the surface of NT-2/D1 and H9 hPSCs. (**a**) 108-B6 and 4A68 recognize approximately 25 kDa cell surface proteins. NT-2/D1 cells were biotinylated, and cell lysates were subjected to immunoprecipitation with 108-B6 or 4A68 and visualized with SA-HRP. Immunoprecipitated proteins (approximately 25 kDa) are indicated by an arrowhead. Con represents immunoprecipitation with Protein G-Sepharose beads alone. (**b**) 108-B6 and 4A68 recognize the same PGRMC1 protein. NT-2/D1 cell lysates were immunoprecipitated with 108-B6 or 4A68. The immunoprecipitated proteins were analyzed by Western blot with 108-B6 or α-PGRMC1 followed by incubation with HRP-conjugated anti-mouse IgG or HRP-conjugated anti-rabbit IgG, respectively. The monomer (M) and trimer (T) forms of PGRMC1 were detected and indicated by arrowheads (top panel). HC represents immunoglobulin heavy chain. Full-length blots are presented in Supplementary Figure 8. (**c**) 108-B6 and α-PGRMC1 recognize the same PGRMC1 protein in hPSCs. H9 hPSC lysates were immunoprecipitated with 108-B6 and α-PGRMC1, and the immunoprecipitated proteins were detected by Western blot with α-PGRMC1 or 108-B6 followed by incubation with HRP-conjugated rabbit IgG or HRP-conjugated mouse IgG, respectively. Full-length blots are presented in Supplementary Figure 8. (**d**) Overexpression and detection of PGRMC1 with 108-B6, 4A68 or α-PGRMC1. HEK293T cells were transfected with pcDNA3.1+ (vector), pCMV-SPORT6-HSPA8 (irrelevant plasmid) or pCMV-SPORT6-PGRMC1 (PGRMC1). The cell lysates were subjected to Western blot with 108-B6, 4A68, or α-PGRMC1. The monomer (M) and trimer (T) forms of PGRMC1 were detected and indicated by arrowheads (top panel). β-actin was used as internal protein control and loading control. Full-length blots are presented in Supplementary Figure 8. In (**a**–**d**), images are representative of at least two independent experiments. (**e**) Flow cytometric analysis of H9 hPSCs with 108-B6, 4A68, and α-PGRMC1.

To identify the cell surface antigens recognized by 108-B6 and 4A68, surface proteins of NT-2/D1 cells were biotinylated, and the cell lysates were immunoprecipitated with 108-B6 and 4A68. The immunoprecipitates were then visualized with streptavidin-horseradish peroxidase (SA-HRP). Both MAbs recognized approximately 25 kDa surface proteins on NT-2/D1 cells (Fig. 2a). The same proteins were excised and subjected to mass spectrometry, and were identified as PGRMC1 from a protein database search (Supplementary Fig. 2a,b). To confirm whether both MAbs recognize PGRMC1, NT-2/D1 cell lysates were immunoprecipitated with 108-B6 and 4A68, and the immunoprecipitates were detected by Western blotting with 108-B6 or a commercially available polyclonal anti-PGRMC1 antibody (α-PGRMC1). The 25 kDa proteins were detected in both immunoprecipitates with 108-B6 and α-PGRMC1 (Fig. 2b), showing that the 108-B6 and 4A68 antigens are the same PGRMC1 indeed. Both 108-B6 and 4A68 were also able to recognize proteins with approximately 66 kDa, which represents a trimer form (T) of PGRMC1^21^ (Fig. 2b, top panel). When H9 hPSC lysates were immunoprecipitated and Western-blotted with 108-B6 and α-PGRMC1, the same 25 kDa proteins were simultaneously detected with 108-B6 or α-PGRMC1 as well (Fig. 2c). To further demonstrate that 108-B6 and 4A68 recognize PGRMC1, irrelevant and PGRMC1 expression plasmids were transfected into HEK293T cells, and the cell lysates were subjected to Western blotting with 108-B6, 4A68 and α-PGRMC1. Overexpressed PGRMC1 proteins were only detected in PGRMC1-transfected lysates with all three antibodies (Fig. 2d). The trimer form of PGRMC1 was also detected in PGRMC1-transfected lysates with 108-B6 (Fig. 2d, top panel). The results confirmed again that 108-B6 and 4A68 antigens are PGRMC1 indeed.

108-B6- and 4A68-reactive PGRMC1 were detected on the surface of various hPSCs including H9, H1, CHA-hES4, NT-2/D1 and NCCIT (Table 1). To confirm whether PGRMC1 is also expressed on the cell surface of hPSCs, propidium iodide (PI)-negative live H9 hPSCs were stained with 108-B6, 4A68, and α-PGRMC1 in flow cytometric analysis. As shown in Fig. 2e, α-PGRMC1 was able to detect PGRMC1 on the surface of hPSCs as 108-B6 and 4A68 did. Taken together, the results indicate that cell surface-expressed PGRMC1 is the target antigen of 108-B6 and 4A68, and is predominantly expressed on the surface of hPSCs.

### PGRMC1 is rapidly downregulated during early differentiation of hPSCs

**Figure 3:**
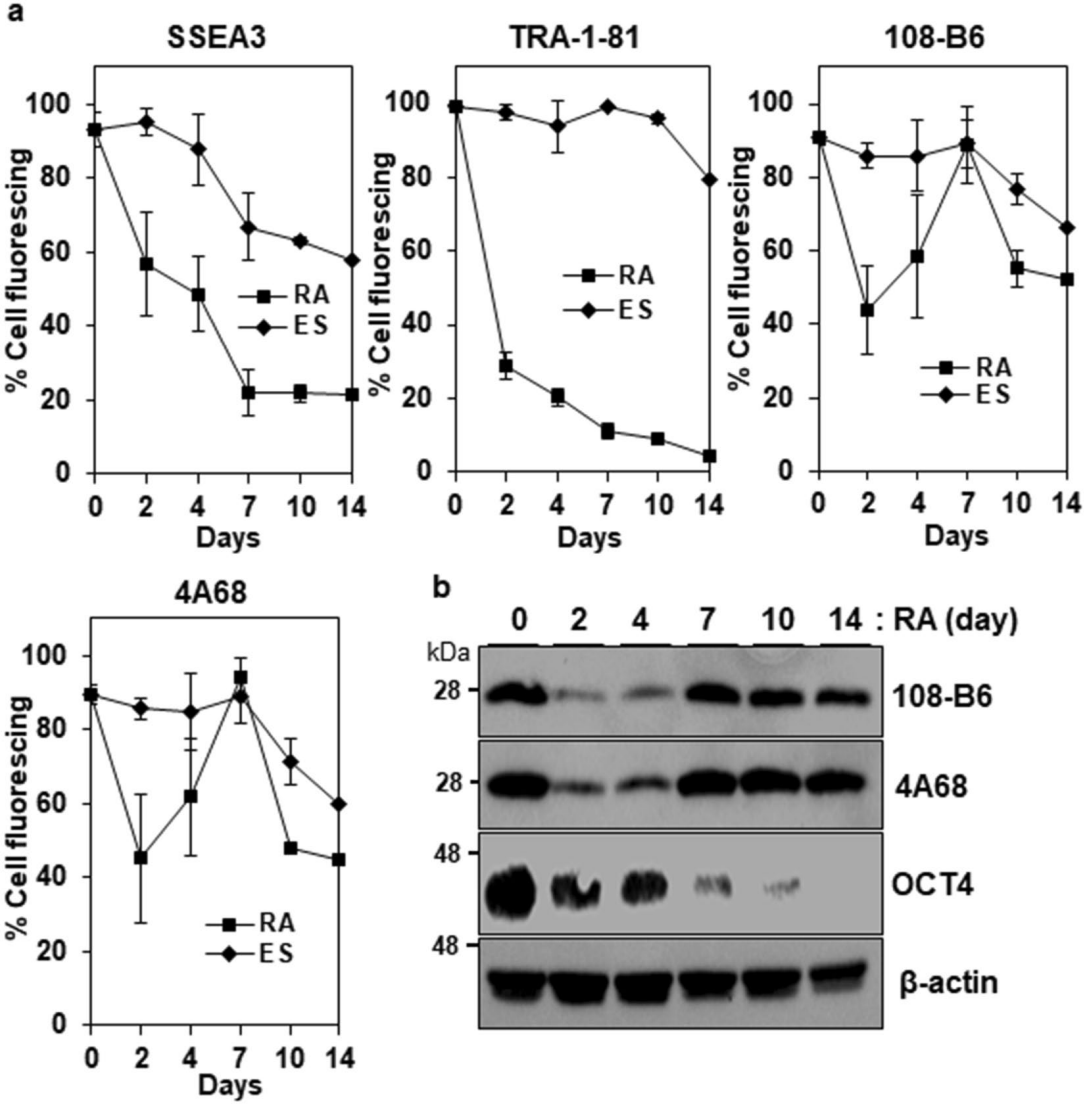
Expression of 108-B6 and 4A68 antigens upon differentiation. (**a**) Flow cytometric analysis of the binding percentages of anti-TRA-1-81, anti-SSEA3, 108-B6 or 4A68 antibodies to undifferentiated H9 hPSCs (ES) or retinoic acid-treated H9 cells (RA). Undifferentiated H9 hPSCs were cultured in the presence of RA (10^−5^ M) for 14 days. ES or RA cells were incubated with anti-TRA-81, anti-SSEA3, 108-B6 or 4A68 antibodies and further incubated with appropriate FITC–conjugated secondary antibodies. The percentages of antibody bindings were calculated by dividing mean fluorescence indexes (MFIs) of antibody bindings with the MFIs of antibody bindings at day 0 (*n* = 3). Error bars indicate standard deviations (SDs) and n is the number of experiments. (**b**) Western blot analysis of differentiated hESCs with 108-B6, 4A68 or anti-OCT4 antibodies. The cell lysates from RA-treated H9 cells were run on a 12% SDS-gel and analyzed with 108-B6, 4A68, anti-OCT4 or β-actin, followed by incubation with HRP-conjugated secondary antibodies. β-actin was used as internal control. Full-length blots are presented in Supplementary Figure 8. Images are representative of at least two independent experiments.

To study whether the expression of PGRMC1 was altered during differentiation of hPSCs, the expression of 108-B6-reactive PGRMC1 was estimated during retinoic acid (RA) treatment by flow cytometry. As with TRA-1-81 and SSEA-3, the expression of 108-B6-reactive PGRMC1 was rapidly down-regulated until 4 days of differentiation (Fig. 3a). However, it was upregulated at 7 days of differentiation, and was gradually downregulated again after 7 days of differentiation (Fig. 3a). Similar results were also obtained with 4A68-reactive PGRMC1, and the similar result were also confirmed by Western blot analysis (Fig. 3b). Thus, PGRMC1 is rapidly downregulated during early differentiation of hPSCs, although it is upregulated later.

### PGRMC1 is required for maintaining hPSC self-renewal without affecting the expression of pluripotency markers

**Figure 4:**
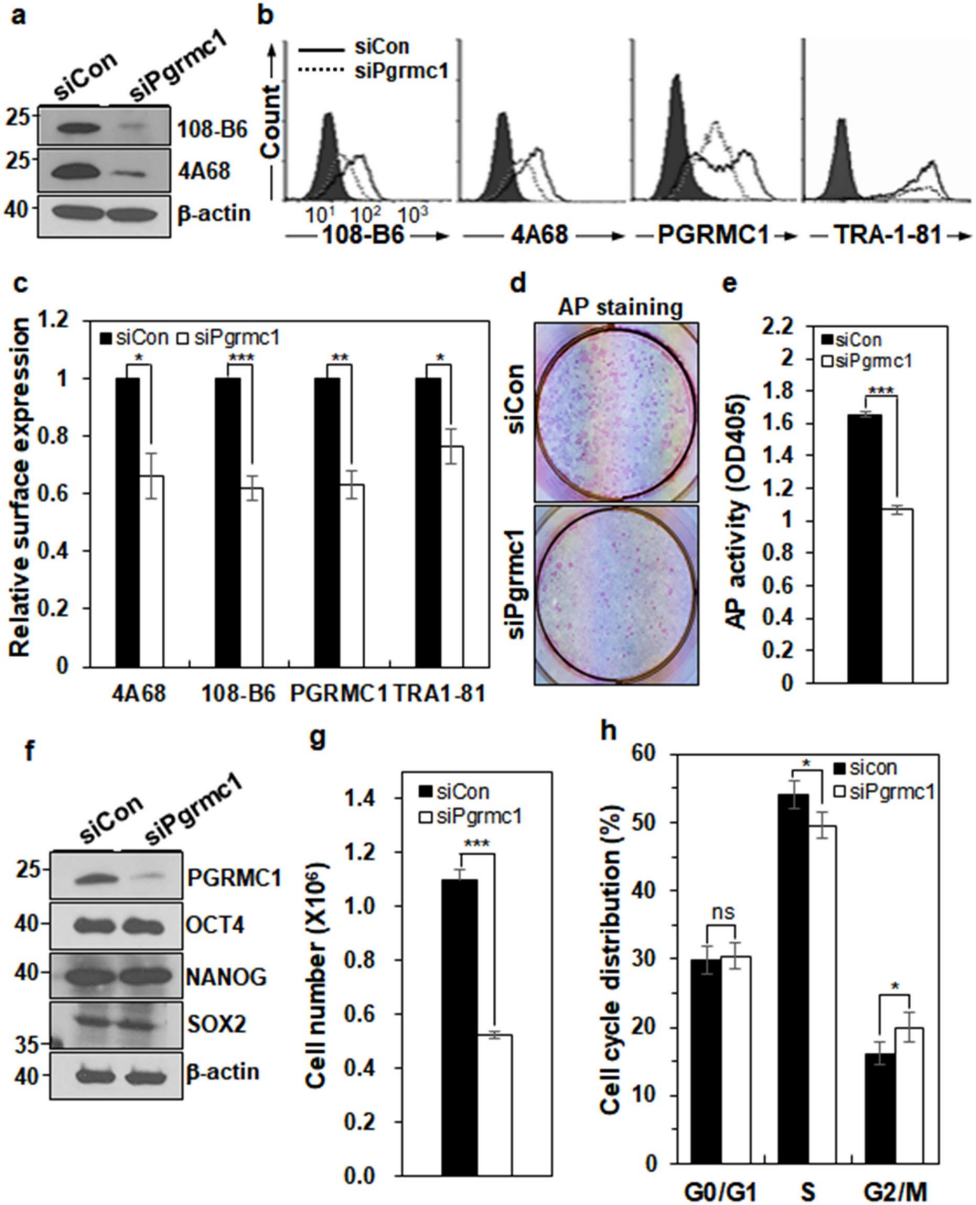
PGRMC1 knockdown impairs hPSC self-renewal and proliferation. (**a**) Knockdown of PGRMC1 in H9 hPSCs. H9 cells were transfected with either control siRNA (siCon) or PGRMC1 siRNA (siPgrmc1) for 72 h, and subjected to Western blot analysis with 108-B6 and 4A68, followed by HRP-conjugated anti-mouse IgG. Full-length blots are presented in Supplementary Figure 8. (**b**) Flow cytometric analysis of cell surface PGRMC1 and TRA-1-81 on the surface of H9 cells transfected with siCon or siPgrmc1. Transfected-H9 cells were incubated with 108-B6, 4A68, α-PGRMC1 or anti-TRA-1-81 antibodies, and detected by appropriate FITC-conjugated secondary antibodies. (**c**) Graphic presentation of cell surface expression of PGRMC1 and TRA-1-81 in siCon or siPgrmc1 knockdown hESCs. Relative expression of cell surface PGRMC1 and TRA-1-81 was measured by MFIs of flow cytometry and normalized for control secondary antibody binding. The graph represents the mean values of three independent determinations ±SD (n = 3; *p < 0.05; **p < 0.01; ***p < 0.005). (**d**) AP staining of siCon or siPgrmc1 knockdown hPSCs. H9 cells were pre-treated with Y-27632, dissociated into single cells, and transfected with siCon or siPgrmc1. After culturing for 7 days, visible colonies were stained with AP assay kit. (**e**) Statistical analysis of AP activity of control or PGRMC1 knockdown hPSCs. AP activity of AP-positive colonies was measured at OD405 (n = 7; ***p < 0.005). (**f**) Western blot analysis showing the expression levels of OCT4, NANOG, SOX2, and PGRMC1 in control or PGRMC1 knockdown hPSCs. β-actin was used as internal protein control and loading control. Full-length blots are presented in Supplementary Figure 8. (**g**) Cell proliferation of control or PGRMC1 knockdown hESCs. Cell numbers of control or PGRMC1 knockdown hPSCs were determined by trypan blue exclusion assay (n = 7; ***p < 0.005). (**h**) Cell cycle distribution of control or PGMC1 knockdown hPSCs. Each bar represents mean values of four independent experiments ± SD (n = 4; *p < 0.05; ns, not significant). In (**a**,**f**), images are representative of at least three independent experiments.

To understand the biological function of PGRMC1 in hPSCs, the PGRMC1 gene was silenced using small interfering RNA in H9 hPSCs. PGRMC1 protein expression was decreased by approximately 79–91%, depending on the experimental conditions (Fig. 4a and Supplementary Fig. 3a). Flow cytometric analysis also confirmed that cell surface-expressed PGRMC1 was decreased by approximately 34–37% in PGRMC1 knockdown hPSCs, while the expression of TRA-1-81 was decreased by approximately 24% in PGRMC1 knockdown hPSCs (Fig. 4b,c). PGRMC1 knockdown hPSCs showed a flat and spread-out morphology (Supplementary Fig. 3b). The colony forming assay showed that alkaline phosphatase (AP) activity was decreased in PGRMC1 knockdown hPSCs (Fig. 4d,e), suggesting that PGRMC1 is necessary for hPSC self-renewal. Interestingly, the expression of pluripotency markers OCT4, SOX2, and NANOG was not significantly altered in PGRMC1 knockdown hPSCs (Fig. 4f). Immunocytochemical analysis also showed that the expression of OCT4, SOX2, and NANOG was not significantly altered in PGRMC1 knockdown hPSCs (Supplementary Fig. 4). As expected, cell proliferation was also decreased by approximately 52% (*p* < 0.005) in PGRMC1 knockdown hPSCs (Fig. 4g). Cell cycle analysis showed a slight decrease (3.2–5.5%) in the percentage of cells in S phase, with a slight increase (2.9–4.7%) in G2/M phase (Fig. 4h), which is consistent with the previous study^22^. Thus, the results suggest that PGRMC1 is required for maintaining hPSC self-renewal and proliferation without affecting the expression of pluripotency markers.

### PGRMC1 maintains hPSC pluripotency through prevention of multi-lineage differentiation of hPSCs

**Figure 5:**
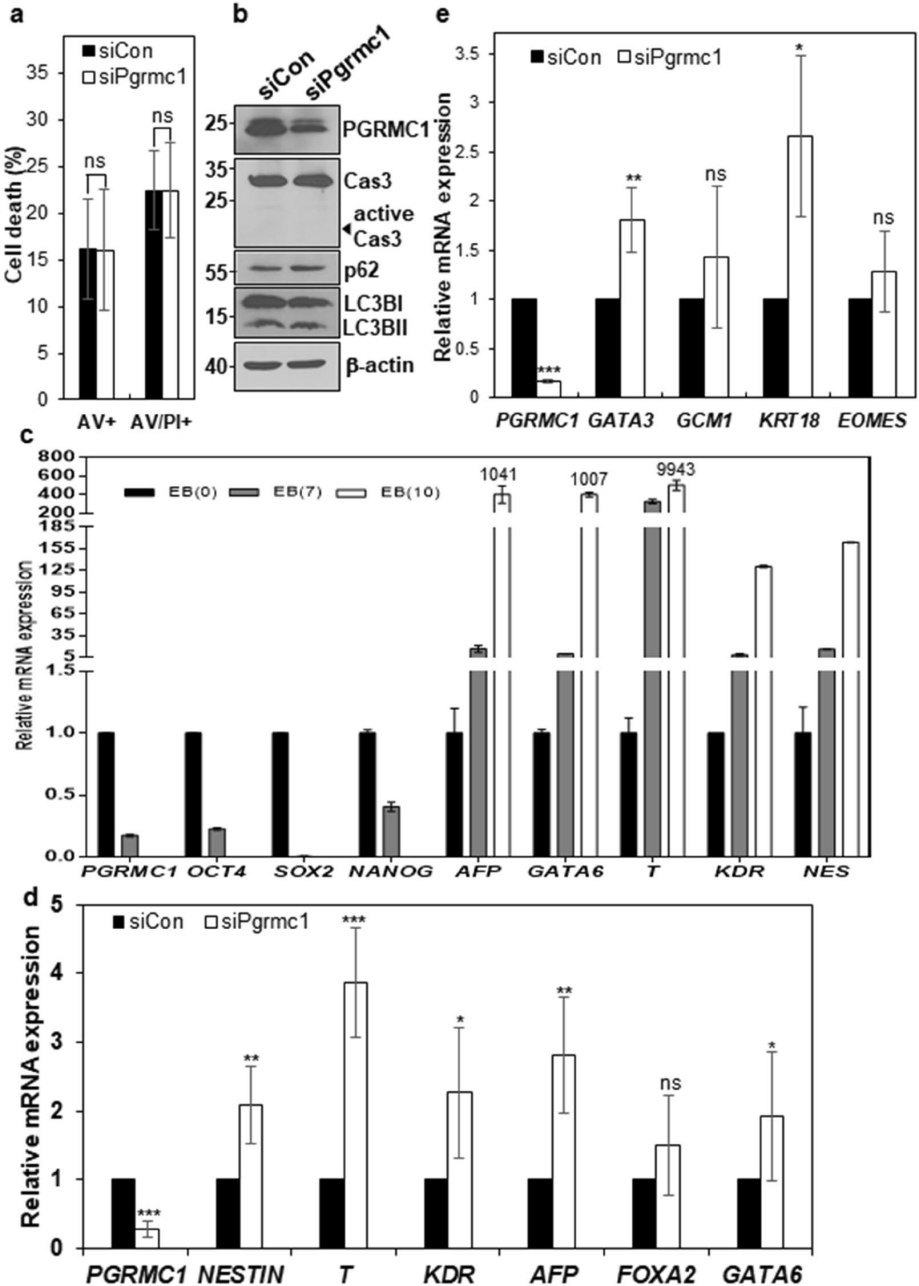
PGRMC1 knockdown drives differentiation of hPSCs into multi-lineage cells. (**a**) Flow cytometric analysis of early and late apoptotic cells with annexin V and PI. Control or PGRMC1 knockdown H9 hPSCs were stained with PI and annexin V-FITC. Shown is statistical analysis of single annexin V-positive (AV+) and both annexin V- and PI-positive cells (AV+PI+). Each bar represents the mean value of three independent experiments ± SD (n = 4; ns, not significant). (**b**) The expression levels of caspase-3, active caspase-3, p62 and LC3B in control and PGRMC1 knockdown hPSCs. β-actin was used as internal protein control and loading control. Full-length blots are presented in Supplementary Figure 8. Images are representative of at least three independent experiments. (**c**) Real-time PCR analysis of mRNA levels of early differentiation genes in EB of hPSCs. EBs were cultured for 7 and 10 days. The graph represents the mean values of two independent determinations ± SD. (**d**) Real-time PCR analysis of mRNA levels of early differentiation genes in control or PGRMC1 knockdown hPSCs. The graph represents the mean values of seven independent determinations ±SD (n = 7; *p < 0.05; **p < 0.01; ***p < 0.005). (**e**) Real-time PCR analysis of mRNA levels of trophectoderm genes in control or PGRMC1 knockdown hPSCs. The graph represents the mean values of 5 independent determinations ± SD (n = 5; ***p < 0.005; ns, not significant).

To investigate whether PGRMC1 knockdown induces apoptosis in hPSCs, control and PGRMC1 knockdown H9 hPSCs were stained with PI and fluorescein isothiocyanate (FITC)-conjugated annexin V. The proportion of early and late apoptotic cells was not changed in PGRMC1 knockdown hPSCs (Fig. 5a and Supplementary Fig. 5a). Real-time PCR analysis also showed that there was no increase in pro-apoptotic and anti-apoptotic markers, although BID alone was slightly increased in PGRMC1 knockdown hPSCs (Supplementary Fig. 5b). As expected, active caspase 3 was not detected in PGRMC1 knockdown hPSCs (Fig. 5b). To further confirm whether PGRMC1 regulates cell death in hPSCs, the markers of autophagic influx were also examined by Western blot analysis because PGRMC1 promotes autophagy in cancer cells^7^. The levels of LC3BII and p62, representative markers of autophagic influx, were not altered in PGRMC1 knockdown hPSCs (Fig. 5b). The results suggest that the modulation of hPSC self-renewal and pluripotency by PGRMC1 is not based upon its inhibition effects on apoptosis and autophagy.

To investigate whether PGRMC knockdown affects the early differentiation state of hPSCs, we examined the expression of representative markers of three germ layers and trophectoderm by realtime PCR in PGRMC1 knockdown hPSCs. Embryoid bodies (EB) were also induced from hPSCs and included in the analysis as a control. All differentiation genes were increased by more than 8-fold at days 7 of EB differentiation and were dramatically increased at days 10 of EB differentiation (Fig. 5c). Among 11 genes of three germ layers and trophectoderm, *NESTIN* (ectoderm), *Brachyury T, kinase insert domain receptor (KDR)* (mesoderm), *α-fetoprotein* (*AFP), GATA6* (endoderm), *GATA3*, and *keratin 18 (KRT18)* (trophectoderm) were increased by approximately 1.8~3.9-fold in PGRMC1 knockdown hPSCs (Fig. 5d,e). Thus, PGRMC1 maintains hPSC pluripotency through the prevention of multi-lineage differentiation of hPSCs.

### PGRMC1 suppresses cyclin D1 expression and p53-dependent pathway in hPSC

**Figure 6:**
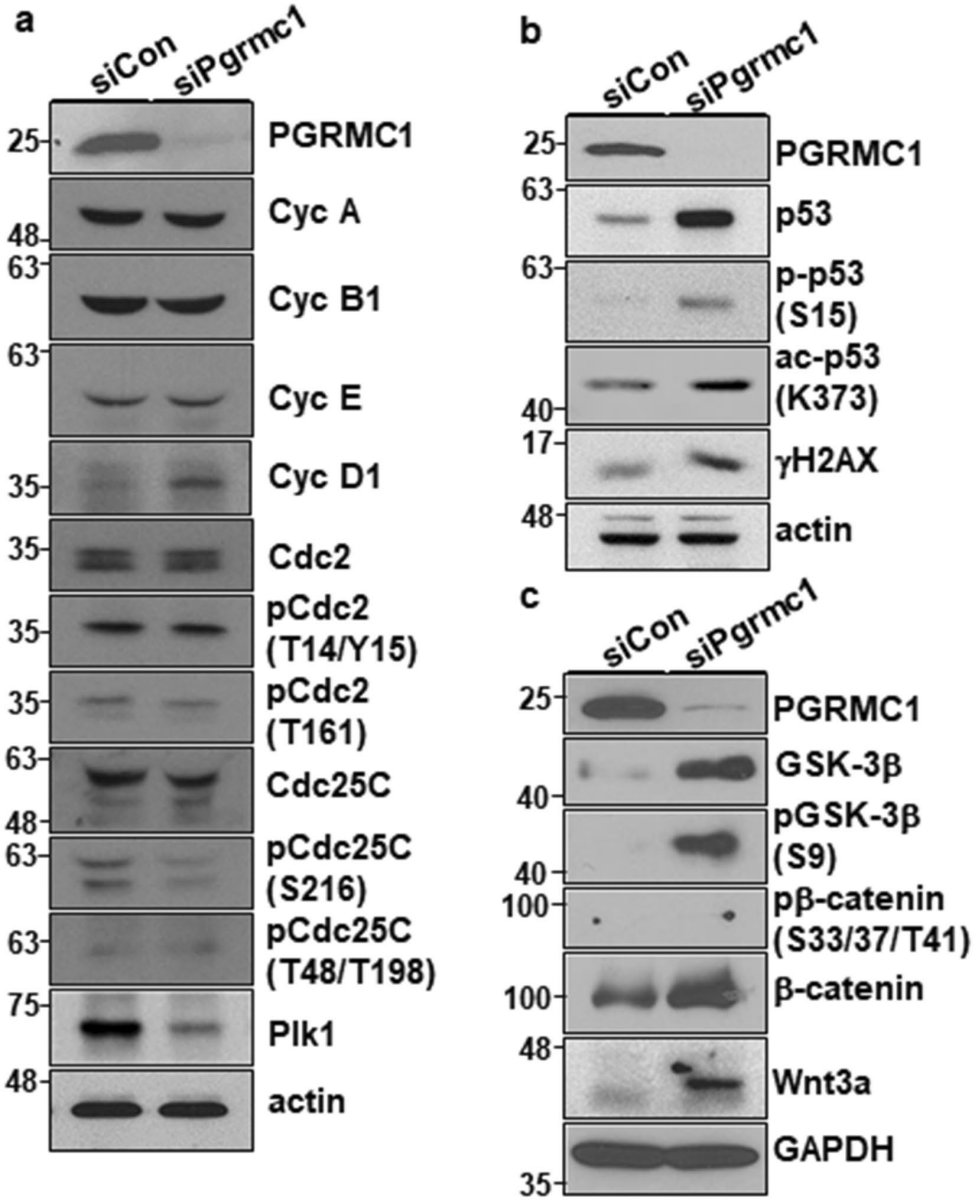
PGRMC1 knockdown increases cyclin D1 and p53 expression, inhibits GSK-3β signaling, and activates β-catenin signaling. (**a**) Expression and phosphorylation analysis of cell cycle regulators and p53 in control or PGRMC1 knockdown hPSCs. Cell lysates were analyzed by Western blot analysis with indicated antibodies. Actin was used as internal protein control and loading control. Full-length blots are presented in Supplementary Figure 9. (**b**) Expression, phosphorylation, and acetylation analysis of PGRMC1, p53, and/or γH2AX in control or PGRMC1 knockdown hPSCs. Cell lysates were analyzed by Western blot analysis with indicated antibodies. Actin was used as internal protein control and loading control. Full-length blots are presented in Supplementary Figure 9. (**c**) Expression and phosphorylation analysis of PGRMC1, GSK-3β, β-catenin, and Wnt3a in control or PGRMC1 knockdown hPSCs. Cell lysates were analyzed by Western blot analysis with indicated antibodies. GAPDH was used as internal protein control and loading control. Full-length blots are presented in Supplementary Figure 9. In (**a**–**c**), images are representative of at least two independent experiments.

PGRMC1 knockdown studies revealed that PGRMC1 regulates hPSC differentiation (Fig. 5d,e). Previous studies have shown that cyclin D1 overexpression controls cell fate decisions in hPSCs by recruiting transcriptional corepressors and coactivator complexes onto neuroectoderm, mesoderm, and endoderm genes^23,24^. Interestingly, PGRMC1 knockdown increased the expression of cyclin D1 in hPSCs, although it did not induce significant alterations in the expression of cyclin A, cyclin B1 and cyclin E (Fig. 6a). The results suggest that PGRMC1 inhibits hPSC differentiation through suppression of cyclin D1 expression.

PGRMC1 inhibition increases the percentage of cells in G2/M phase in cultured bovine granulosa cells and maturing oocytes^22^. The present study also found that PGRMC1 knockdown caused G2/M cell cycle arrest (Fig. 4h). Furthermore, PGRMC1 knockdown caused large-sized nuclei and micronuclei in hPSCs, as compared with control knockdown hPSCs (Supplementary Fig. 4). In the analysis of cell cycle regulators, PGRMC1 knockdown did not induce alterations in the phosphorylation of the core mitotic regulators cell division cycle 2 (Cdc2) and cell division cycle 25C (Cdc25C) in hPSCs (Fig. 6a). However, PGRMC1 knockdown induced decreased expression of polo-like kinase 1 (Plk1) (Fig. 6a), a critical mediator of G2/M cell cycle transition, suggesting that PGRMC1 knockdown reduces the mitotic activity of hPSCs through downregulation of Plk1.

Interestingly, PGRMC1 knockdown increased p53 and γH2AX (H2A histone family, member X) expression in hPSCs (Fig. 6b). The phosphorylation of p53 at serine 15 was increased in PGRMC1 knockdown hPSCs, and the acetylation of p53 at lysine 373 was also increased in PGRMC1 knockdown hPSCs (Fig. 6b), suggesting that PGRMC1 suppresses p53 expression and stability in hPSCs. p53 expression and stability regulate cell cycle to promote differentiation of hESCs into diverse lineage cells^25,26^. Previous studies also have shown that p53 represses Plk1 expression^27,28^, which is consistent with the present study. Therefore, the results suggest that PGRMC1 suppresses p53 expression and stability to maintain hPSC self-renewal and pluripotency.

### PGRMC1 inhibits expression of CycD1 and differentiation genes in hPSCs directly or through suppression of Wnt/β-catenin pathway

To further gain insight into the mechanism of how PGRMC1 regulates hPSC self-renewal and pluripotency, we analyzed the key signaling molecules on hPSC self-renewal and differentiation. However, PGRMC1 knockdown did not induce any changes in the phosphorylation of Akt1/2//3 and S6 in hPSCs (Supplementary Fig. 6a,b). PGRMC1 knockdown did not change the phosphorylation of extracellular signal-regulated kinase (ERK1/2) either (Supplementary Fig. 6a,b), suggesting that PGRMC1 is not associated with Akt1/2/3 and ERK1/2 signaling. Instead, PGRMC1 knockdown caused increased expression of glycogen synthase kinase 3 (GSK-3β) (Fig. 6c). Interestingly, inhibitory phosphorylation of GSK-3β at serine 9 was drastically increased in PGRMC1 knockdown hPSCs as well (Fig. 6c). As expected, enhanced expression of β-catenin was observed in PGRMC1 knockdown hPSCs, and the phosphorylation of β-catenin at S33, 37, and T41 was not detected (Fig. 6c), suggesting that GSK-3β is inactivated, and active β-catenin, a key downstream effector of GSK-3β, is increased in PGRMC1 knockdown hPSCs. Furthermore, PGRMC1 knockdown increased Wnt3a expression in hPSCs (Fig. 6c), suggesting that PGRMC1 inhibits Wnt/β-catenin signaling in hPSCs. To further confirm whether PGRMC1-dependent inhibition of Wnt/β-catenin signaling is due to off-target effects of PGRMC1 siRNA, PGRMC1 was also knocked down by two additional PGRMC1 siRNAs (#2 and #3). Again, PGRMC1 knockdown increased Wnt3a and β-catenin expression in hPSCs, although the degree of increase was slightly different between siRNAs used (Supplementary Fig. 6c,d). To avoid extra discussion of cell line-specific function of PGRMC1 in hPSCs, we also analyzed the expression of *Wnt3a, CycD1* and differentiation genes in PGRMC1-transfected CHA-hES4^29^ and obtained similar results (Supplementary Fig. 7). Taken together, the results suggest that PGRMC1 regulates hPSC self-renewal and pluripotency through suppression of Wnt/β-catenin signaling.

**Figure 7:**
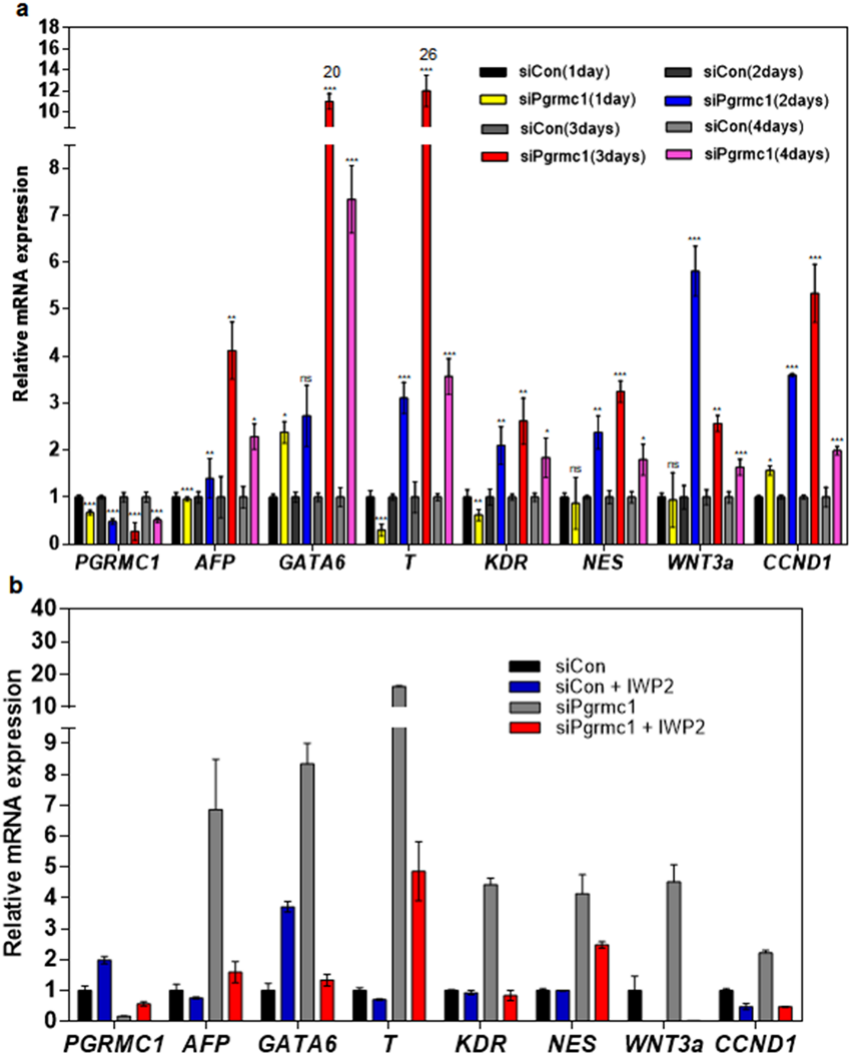
PGRMC1 inhibits differentiation genes and *CycD1* in hPSCs directly or via suppression of Wnt/β-catenin pathway. (**a**) Real-time PCR analysis of mRNA levels of *Wnt3a, CycD1* and early differentiation genes in control or PGRMC1 knockdown H9 hPSCs. The graph represents the mean values of four independent determinations ± SD (n = 4; *p < 0.05; **p < 0.01; ***p < 0.005; ns, not significant). (**b**) Real-time PCR analysis of mRNA levels of *Wnt3a, CycD1* and early differentiation genes in control or PGRMC1 knockdown H9 hPSCs after IWP-2 treatment. The graph represents the mean values of 2 independent determinations ± SD.

However, it is also possible that Wnt3a may be induced after differentiation genes induced by other pathways. To examine whether *Wnt3a* expression precedes differentiation gene expression, we did a time course study (1~4 days) showing the expression of *Wnt3a, CycD1* and differentiation genes in PGRMC1 knockdown hPSCs (Fig. 7a). *Wnt3a* expression was obviously increased on day 2 post-transfection in PGRMC1 knockdown hPSCs. Differentiation genes *AFP, T, KDR* and *NES* were also significantly increased on day 2 post-transfection in PGRMC1 knockdown hPSCs. However, *GATA6* and *CycD1* were increased on day 1 post-transfection before *Wnt3a* expression. The results suggest that Wnt/β-catenin signaling does not precede at least *GATA6* and *CycD1* expression. The results further suggest that PGRMC1 directly regulates *GATA6* and *CycD1* genes. To further figure out the correlation between Wnt3a and differentiation genes, PGRMC1 knockdown hPSCs were treated with IWP-2, an inhibitor of Wnt processing and secretion, on day 1 post-transfection (Fig. 7b). IWP-2 treatment drastically decreased *Wnt3a* expression in PGRMC1 knockdown hPSCs. All differentiation genes including *GATA6* were also decreased in IWP-2-treated PGRMC1 knockdown hPSCs. Interestingly, *CycD1* gene was also decreased in IWP-2-treated PGRMC1 knockdown hPSCs. The results suggest that Wnt/β-catenin could be an upstream regulator of all differentiation genes and CycD1 in PGRMC1 knockdown hPSCs. Taken together, the results suggest that PGRMC1 inhibits expression of cyclin D1 and differentiation genes in hPSCs directly or through suppression of Wnt/β-catenin pathway.

## Discussion

PGRMC1 is a multi-functional protein that is induced in various cancers and is critical for tumor growth, invasion and metastasis^1^. PGRMC1 is mainly localized in the endoplasmic reticulum, mitochondria, nucleus membrane, and nucleolus in multiple cancer cells^1,30^. However, some studies have also suggested that PGRMC1 is expressed on the surface of cancer and neuronal cells^5,15,31^. The present study also found that PGRMC1 was expressed on the surface of hPSCs, and cell surface-expressed PGRMC1 was rapidly downregulated during early differentiation of hPSCs (Fig. 2), which led us to study the role of PGRMC1 in hPSC self-renewal and pluripotency. PGRMC1 knockdown experiments demonstrated that PGRMC1 promotes self-renewal and inhibits differentiation in hPSCs through suppression of Wnt/β-catenin signaling pathway. Interestingly, PGRMC1 regulates hPSC self-renewal and pluripotency without affecting the expression of pluripotency markers (Figs 4f and 5d,e). Studies have shown that some factors regulate PSC self-renewal and pluripotency without affecting the expression levels of pluripotency markers. Banf1 knockdown disrupts the self-renewal and survival of hESCs without reducing the expression levels of OCT4, SOX2 and NANOG^32^. Msi2 knockdown also causes mESC to differentiate despite continued expression of both SOX2 and OCT4^33^. Rem2 GTPase knockdown also impairs hESC self-renewal and pluripotency without affecting the expression of main pluripotency markers^34^. Nucleolin knockdown also leads to mESC differentiation, as well as decreased self-renewal ability without significant alterations in the expression of pluripotency markers^35^. Studies showed that PGRMC1 is expressed in the nucleolus and colocalized with nucleolin (http://www.proteinatlas.org/search/pgrmc1)^36^. Furthermore, it appears that nucleolin maintains self-renewal of mESCs by suppression of p53-dependent pathway^35^, which is consistent with the function of PGRMC1 in the present study (Fig. 6b). Therefore, it is tempting to speculate that PGRMC1 may be functionally associated with nucleolin in hPSCs. More extensive investigations of the relationship between PGRMC1 and nucleolin in hPSCs could verify the above speculation.

**Figure 8:**
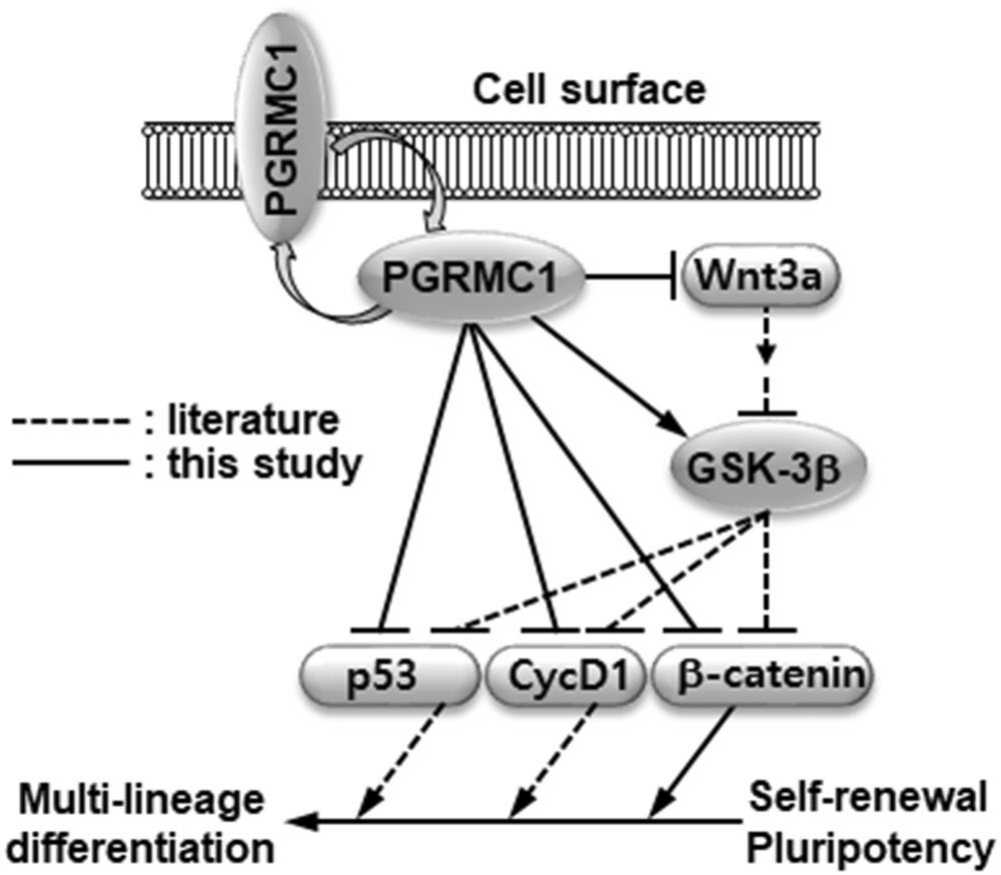
Proposed model for the function of PGRMC1 in hPSCs. PGRMC1 is expressed on the cell surface and inside the cell. PGRMC1 directly suppresses CycD1, p53 and β-catenin and indirectly suppresses them through suppression of Wnt/β-catenin signaling. PGRMC1 also suppresses β-catenin through activation of GSK-3β or inhibition of Wnt3a expression. PGRMC1-mediated suppression of p53, cyclin D1, and β-catenin inhibits differentiation of hPSCs into multi-lineage cells during early differentiation of hPSCs.

The present study found that PGRMC1 regulates early differentiation of hPSCs. Actually, a previous study reports that PGRMC1 is expressed in a variety of primary stem cells including hESCs, and the expression of this protein depends on the state of stem cell differentiation^19^, which suggests that PGRMC1 may be an important regulator of stem cell differentiation. In an attempt to gain insight into the underlying mechanism of how PGRMC1 regulates hPSC differentiation, we found that PGRMC1 knockdown increased Wnt3a and β-catenin expression in hPSCs (Fig. 6c). PGRMC1 knockdown also increased inhibitory phosphorylation of GSK-3β (Fig. 6c). The results suggest that PGRMC1 inhibits hPSC differentiation through suppression of Wnt/β-catenin signaling. PGRMC1 knockdown also increased the expression of cyclin D1 and induced p53 expression and stabilization in hPSCs (Fig. 6a,b). Studies have shown that activation of the Wnt/β-catenin signaling drives the differentiation of hPSCs into mesendodermal lineage cells^37,38^. Increased expression of cyclin D1 also drives the differentiation of hPSCs into neuroectodermal lineages^23,24^. p53 expression and stabilization also lead to expression of endoderm markers GATA4 and AFP, as well as ectoderm marker PAX6 in hPSCs^26^. Therefore, it seems that PGRMC1 is able to suppress broad networks necessary for multi-lineage fate specification in hPSCs through suppression of Wnt/β-catenin, cyclin D1, and p53-associated pathways. Inhibition of GSK-3β rescues p53 from degradation^39,40^. GSK-3β promotes cyclin D1 proteolysis^41^. Therefore, the increased expression of cyclin D1 and p53 is consistent with the enhanced inhibitory phosphorylation of GSK-3β at serine 9 in PGRMC1 knockdown hPSCs. Based on the previous studies that Wnt/GSK-3β/β-catenin signaling is able to regulate the expression of cyclin D1 and p53^39–41^, a proposed model for the function of PGRMC1 in hPSC self-renewal and pluripotency is presented (Fig. 8).

The functional mechanism described above is a non-genomic mechanism of action for PGRMC1. However, it is also possible that PGRMC1 directly regulates cell fate decision by binding to differentiation genes as a transcriptional repressor or corepressor. This notion is based on the concept that PGRMC1 influences gene transcription. In this regard, it is interesting to note that progesterone-dependent PGRMC1 suppresses the transcriptional activity of T-cell factor/lymphoid enhancer factor (Tcf/Lef)^42,43^. β-catenin/Tcf/Lef is the representative transcriptional regulatory pathway of Wnt/β-catenin signaling^44^. Therefore, it is also tempting to speculate that PGRMC1 is able to directly suppress the expression of differentiation genes in a progesterone-dependent manner. The underlying mechanism of how PGRMC1 regulates hPSC differentiation genomically or non-genomically remains to be further studied.

PGRMC1 knockdown induced p53 expression, activation, and stabilization in hPSCs (Fig. 6b). The next interesting question is how PGRMC1 induces p53 expression and stability in hPSCs. First, the increased stability of p53 in PGRMC1 knockdown hPSCs may be due to the inhibitory phosphorylation of GSK-3β, because studies have shown that inhibition of GSK-3β rescues p53 from degradation^39,40^. Second, the increased stability of p53 may be due to the possible absence of nuclear translocation of heme in PGRMC1 knockdown hESCs, because PGRMC1 may play in transferring heme between suborganelles^30^. This speculation is based on studies showing that heme directly binds to p53 protein and promotes nuclear export of p53 and its subsequent cytosolic proteolysis^45,46^. Third, p53 induction may be due to the damage responses induced by unfaithful chromosomal segregation shown in PGRMC1 knockdown hPSCs (Supplementary Fig. 4). Supporting this notion, γH2AX induction was observed in PGRMC1 knockdown hPSCs (Fig. 6b). The role of PGRMC1 in chromosomal segregation will be the next interesting research topic in hPSCs.

In summary, MAbs 108-B6 and 4A68 recognized PGRMC1 on the cell surface of hPSCs. Cell surface-expressed PGRMC1 was predominantly expressed on various hPSCs but not on differentiated primary cells. PGRMC1 was rapidly decreased during early differentiation of hPSCs. PGRMC1 knockdown decreased self-renewal and pluripotency of hPSCs without affecting the expression of pluripotency markers. PGRMC1 knockdown did not induce apoptosis and autophagy of hPSCs, but led to differentiation of hPSCs into multiple lineage cells. PGRMC1 knockdown caused increased expression of cyclin D1 and decreased expression of Plk1. PGRMC1 knockdown also induced p53 expression and stability. Analysis of signaling molecules revealed that PGRMC1 knockdown resulted in increased phosphorylation of GSK-3β at serine 9 and increased expression of Wnt3a in hPSCs, leading to inactivation of GSK-3β and activation of Wnt/β-catenin signaling. The results suggest that PGRMC1 suppresses the p53 and Wnt/β-catenin pathways to promote self-renewal and inhibit early differentiation in hPSCs.

## Methods

### Cell culture

hPSC lines (H1 and H9) were cultured on the irradiated mouse embryonic fibroblast (MEF) feeder cells in DMEM/F12 medium (Welgene, Daegu, Korea)^20^, supplemented with 20% serum replacement (Invitrogen, Seoul, Korea), 0.1 mM 2-mercaptoethanol, 1% non-essential amino acids, 32 mM sodium bicarbonate, and 4 ng/ml basic fibroblast growth factor (bFGF) (PeproTech, Rocky Hill, NJ, USA). CHA-hES4 was kindly provided by Dr Hyung Min Chung (CHA University, Seoul, Korea)^29^. Feeder-free cultures of hESCs were grown on Matrigel (BD Biosciences, Seoul, Korea)-coated plates in MEF-conditioned medium containing 12 ng/ml bFGF (PeproTech)^47^ or in TeSR^TM^-E8^TM^ (STEMCELL Technologies, Vancouver, Canada). Differentiation of H9 cells was induced by incorporating all-trans-retinoic acid (RA, Sigma-Aldrich, Seoul, Korea) at 10^−5^ M into the growth medium for at least 14 days. mESC lines R1 (American Type Culture Collection (ATCC), Manassas, VA, USA), and E14Tg2a.4 (Mutant Mouse Regional Resource Center, University of California-Davis, Davis, CA, USA) were cultured as previously described^20^. The human embryonal carcinoma cell lines NT-2/D1 and NCCIT were cultured according to the instructions provided by ATCC. Human PBMCs were isolated by the Ficoll-Paque Plus method (GE Healthcare, Seoul, Korea). HDFs were purchased from MCTT (Seoul, Korea). Cancer cell lines were obtained from ATCC and maintained according to the protocol provided by the supplier.

### EB formation

H9 hPSCs were washed in hPSC medium without bFGF once and then incubate in 3 ml collagenase IV (1 mg/ml) for 5 min at 37 °C to dissociate colonies to cell clumps. Cells were harvested and incubated on gelatin-coated plate in hPSC medium (without bFGF) for 15~30 min to remove MEFs. Cells were collected from the flasks and washed cells again hPSC medium (without bFGF). hPSC clumps were seeded into bacterial dishes via 1:1 ratio and cultured in DMEM/F12 medium supplemented with 20% fetal bovine serum (FBS), 0.1 mM 2-mercaptoethanol, 1% non-essential amino acids, 100 U/ml penicillin G and 100 μg/ml streptomycin for 7 or 10 days. Medium was changed every other day.

### Antibody purification

MAbs were purified from the culture supernatant of hybridomas by Protein G-Agarose column chromatography as described previously^48,49^.

### Flow cytometry and apoptosis

hPSCs, RA-treated hPSCs, mESCs, and various cancer cells and primary cells were harvested as single cell suspensions using using trypsin/EDTA solution (Welgene). The dissociated cells were immediately resuspended in PBA (1% bovine serum albumin, 0.02% NaN_3_ in phosphate buffered saline (PBS), pH 7.4) and incubated with various primary antibodies for 20 min at 4 °C. The cells were then further incubated with FITC-conjugated anti-rat immunoglobulin (Ig) M, anti-mouse IgM, anti-mouse IgG or anti-rabbit IgG (BD Biosciences), depending on the isotypes of the primary antibodies. After washing, PI-negative cells were analyzed for antibody binding using FACS Calibur and Cell Quest software (BD Biosciences). Primary antibodies used were anti-SSEA-3 (R&D System, Minneapolis, MN, USA), anti-TRA-81 (Millipore, Billerica, MA, USA), mouse MAb 108-B6 and 4A68, and anti-PGRMC1 antibody (α-PGRMC1, GeneTex (C2C3), Irvine, CA, USA). For multi-color flow cytometric analysis, cells were probed with 108-B6 or 4A68 followed by incubation with phycoerythrin (PE)-conjugated anti-mouse IgG1 (Thermo Fischer Scientific, Seoul, Korea). The cells were then incubated with anti-SSEA-3 or anti-TRA-1-81. The cells were further incubated with FITC-conjugated anti-rat IgM or anti-mouse IgM (BD Biosciences) before analysis. For intracellular flow cytometric analysis, cells were probed with 108-B6 or 4A68 followed by incubation with PE-conjugated anti-mouse IgG1 (Thermo Fischer Scientific). After wash with PBA, the cells were fixed in 2% paraformaldehyde in PBA for 15 min at 4 °C and permeabilized in PBA containing 0.5% saponin for 15 min at 4 °C. The cells were then incubated with anti-OCT4 (Santa Cruz Biotechnology) or anti-SOX2 (Santa Cruz Biotechnology). The cells were further incubated with Alexa 488-conjugated anti-rabbit IgG (Thermo Fischer Scientific) before analysis. To detect apoptosis, hESCs were stained and analyzed with PI and FITC-conjugated annexin V using an Annexin V-FITC/PI Apoptosis kit (BD Biosciences) according to the manufacturer’s protocol.

### Cell surface biotinylation, immunoprecipitation, and Western blotting

Cell surface biotinylation, immunoprecipitation, and Western blotting were performed as described previously^20,49,50^. Briefly, cell surface biotinylation of NT-2/D1 cells were performed as described in the supplier’s protocol with EZ-Link Sulfo-NHS-LC-Biotin (Thermo Fischer Scientific). Biotin-labeled cells were lysed with 1% NP40 lysis buffer (25 mM Tris-HCl, pH 7.5, 250 mM NaCl, 5 mM EDTA, 0.1% Nonidet P-40, 2 μg/ml aprotinin, 100 μg/ml phenylmethanesulfonyl fluoride, and 2 μg/ml leupeptin) at 4 °C for 30 min. The cell lysates were clarified by centrifugation to remove nuclei at 4 °C, 12000 rpm for 40 min before storage in −70 °C deep freezer. To remove the cellular proteins that nonspecifically bind to Protein G-Sepharose, the cell lysates were incubated with Protein G-Sepharose at 4 °C for 2 h, and the beads were then recovered and extensively washed with lysis buffer for use as a negative control (Con) for immunoprecipitation experiments. For immunoprecipitation of the antigens recognized by the MAbs, the precleared lysates were incubated with approximately 1–2 μg MAbs 108-B6, 4A68 or α-PGRMC1 (GeneTex) at 4 °C overnight and further incubated with Protein G agarose. The beads were extensively washed with lysis buffer, and the bound proteins were eluted from the beads by boiling at 100 °C for 5 min. Proteins were then transferred to a nitrocellulose membrane, and the membrane was blocked in 5% skim milk in PBST (PBS containing 0.1% Tween 20) at room temperature (RT) for 1 h. The membrane was then incubated with SA-HRP (GE Healthcare) at RT for 1 h. After extensive washing, the biotinylated proteins were visualized using enhanced chemiluminescence (ECL) detection reagent (GE Healthcare).

To do Western blotting of various proteins, total extract of various cells was obtained after lysis for 30 min at 4 °C in RIPA buffer (50 mM Tris-HCl, pH 7.4, 150 mM NaCl, 1% NP-40, 0.5% sodium deoxycholic acid, 0.1% SDS). Protein samples were fractionated by sodium dodecyl-sulfate-polyacrylamide gel electrophoresis (SDS-PAGE) on a 12% polyacrylamide gel under denaturing conditions and transferred to a nitrocellulose membrane. The membrane was blocked with 5% skim milk in 0.1% TBST (TBS containing 0.1% Tween 20) at RT for 1 h, and incubated at RT for 2 h with various primary antibodies followed by horseradish peroxidase-conjugated anti-mouse IgG, or anti-rabbit IgG (Millipore). Primary antibodies used were mouse MAbs against PGRMC1, NANOG, β-actin, Akt1/2/3, GSK-3β, ERK1/2, CDC2, (all from Santa Cruz Biotechnology), and S6, cyclin E (Cell Signaling Technology), and rabbit polyclonal antibodies against PGRMC1, OCT4, p-Akt1/2/3, β-catenin, cyclin A, cyclin B1, p-CDC2(T14/Y15), CDC25C (all from Santa Cruz Biotechnology), PGRMC1 (GeneTex), SOX2, Glyceraldehyde-3-phosphate dehydrogenase (GAPDH), Caspase-3 (all from CUSABIO, Wuhan, China), LC3B (Novus Biologicals, Littleton, CO), p62 (Abcam, Cambridge, UK), cyclin D1, p-S6 (S240/244), p-GSK-3β (S9), p-ERK1/2 (T202/Y204), p-β-catenin (S33/37/T41), p-CDC2 (T161), p-CDC25C (S216), p-CDC25C (T48/198), γH2AX (S137), Plk1, p53, p-p53 (S15), p-p53 (S20) (all from Cell Signaling Technology), and anti-acetyl-p53 (K373) (Millipore). The stained bands were visualized by using ECL detection reagent (Advansta, Menlo Park, CA, USA). The signal intensities of Western blots were measured quantitatively using the Image J software. β-actin, actin, or GAPDH were used as a loading control.

### Mass spectrometry

The protein of interest was enzymatically digested in-gel in a manner similar to that described previously^49,51^. The search program ProFound was used for protein identification^52^.

### DNA and siRNA transfection

HEK293T (7 × 10^5^ cells) was transfected with pCMV-SPORT6-PGRMC1 (KRIBB, Daejeon, Korea), pCMV-SPORT6-HSPA8 or pcDNA3.1+ plasmid vector using Lipofectamine™ 2000 (Thermo Fischer Scientific) according to the supplier’s protocol. Protein was transiently expressed for 24 hrs, and the cell pellet was lysed in RIPA buffer. Cell lysates were subjected to Western blot analysis or immunoprecipitation as descried above. To knock PGRMC1 down, H9 or CHA-hES4 cells pre-incubated with 10 μM Y-27632 for 1 h were dissociated with TrypLE Select (Thermo Fisher Scientific), and 2 × 10^5^ cells were seeded into Matrigel (BD Biosciences) coated-feeder free plate in TeSR-E8 medium (STEMCELL Technologies). The next day, the cells were transfected with PGRMC1 siRNA #1 (Ambion, Austin, TX)^3,53^ or accutarget negative control siRNA (Bioneer, Daejeon, Korea) using RNAimax (Thermo Fisher Scientific) according to the supplier’s protocol. PGRMC1 siRNA #2 and #3 were also obtained from Bioneer (Daejeon, Korea) and used to test off-target effects of PGRMC1 siRNAs. The sense sequences of PGRMC1 siRNA #2 and #3 were 5′-CAGUACAGUCGCUAGUCAA-3′ and 5′-CAGUUCACUUUCAAGUAUCA-U-3′, respectively. The same transfection was repeated once after 24 h to increase the transfection efficiency. After transfection, the cells were incubated for 72 h before further analysis.

### Proliferation and alkaline phosphatase assay

To determine the effect of PGRMC1 knockdown in cell proliferation, feeder-free H9 cells were transfected with control or PGRMC1 siRNAs as described above. After 72 h from the transfection, cells were dissociated with 0.05% trypsin-EDTA to determine cell viability by trypan blue exclusion assay. Colony forming assay and AP staining (Cell Biolabs. San Diego, CA, USA) were performed according to manufacturer’s instructions.

### Immunocytochemistry

H9 hPSCs were cultured on MEF feeder cells and stained as described previously^50^. Briefly, H9 hPSCs were fixed with 3.7% paraformaldehyde (PFA) for 15 min, and incubated with 4A68 or 108-B6, and TRA-1-81 antibodies. The cells were then incubated for 1 h at RT in the dark with R-PE-conjugated anti-mouse IgG (Vector Laboratories, Burlingame, CA, USA) and FITC-conjugated anti-mouse IgM (Thermo Fischer Scientific). To detect intracellular antigens, cells were permeabilized with 0.1% Triton X-100 after fixation with 4% PFA, and incubated with anti-OCT4 (Abcam, Cambridge, UK), anti-SOX2 (Abcam), anti-NANOG (Santa Cruz Biotechnology), anti-LC3B (Novus Biologicals), 4A68, 108-B6, and/or anti-PGRMC1 (GeneTex). Cells were then incubated with Alexa 488-conjugated anti-rabbit IgG (Thermo Fischer Scientific) and/or Dylight 649-conjugated anti-mouse IgG (Vector Laboratories). Nuclei were stained with 4′,6-diamidino-2-phenylindole (DAPI). Fluorescence signals were detected with a fluorescence microscope or a Leica TCS SP5 confocal microscope.

### Realtime PCR

H9 or CHA-hES4 hPSCs were transfected with control or PGRMC1 siRNAs as described above. After 24, 48, 72 or 96 h post-transfection, total RNA was extracted as described in the manufacturer’s protocol of RNAiso plus reagent (TaKaRa, Tokyo, Japan). Reverse transcription polymerase chain reaction (RT-PCR, Takara) and real-time PCR (Applied Biosystems, Foster, CA, USA) were performed using the Master Mix kit, according to manufacturer’s instructions. Levels of transcripts for specific genes were determined using specific primers for human transcripts encoding *Wnt3a*^54^*, CycD1, GATA6, Brachyury T*^55^, *KDR, nestin (NES), forkhead box protein A2 (FoxA2), AFP, GAPDH*^32^, *BCL2-associated agonist of cell death (BAD), BCL2 Associated X (BAX), NOXA, B-cell lymphoma 2 (BCL2)*^56^, *BH3 interacting domain death agonist (BID)*^50^ and *PGRMC1*^57^. Each sample was analyzed in triplicate for each gene. The PCR primer sequences for *CycD1* and trophectoderm lineage markers were as follow (forward and reverse, respectively): *CycD1* (5′-TGG AAA CCA TCC GCC GCG C-3′ and 5′-CGA TCT TCC GCA TGG ACG GCA G-3′′), *GATA3* (5′-ACA TGC TGA CCA CGC CCA CG-3′ and 5′-GCA GGG CTC TAA CCC ATG GC-3′), *glial cells missing homolog 1 (GCM1)* (5′-AAG AAG TCC TGC CTG GGT GT-3′ and 5′-CGT GCC TCC AGA AGT TGG T-3′), *KRT18* (5′-GAG TAT GAG GCC CTG CTG AAC ATC A-3′ and 5′-GCG GGT GGT GGT CTT TTG GAT-3′), and *eomesodermin (EOMES)* (5′-AGC TCT CCA AGG AGA AAG TG-3′ and 5′-GCC TTC GCT TAC AAG CAC TG-3′).

### Cell cycle analysis

Feeder-free H9 hPSCs were transfected with control or PGRMC1 siRNAs as described above. After 72 h from the first transfection, H9 hPSCs were treated with 30 μM bromodeoxyuridine (BrdU) for 4 h. Detached cells (0.5 × 10^6^) were fixed in 70% ethanol overnight at 4 °C. After washing with PBS, DNA was denatured by 2 M HCl/0.5% Triton X-100 for 30 min at RT. Cells were then neutralized with 0.1 M sodium borate (pH 8.0) for 2 min. After washing with PBS (pH 7.4), cells were incubated with Alexa 488-conjugated BrdU antibody (Thermo Fisher Scientific) for 30 min at RT and incubated in RNase A (10 μg/ml) and PI solution for 30 min at RT. Finally, cells were analyzed using FACSCalibur (BD Biosciences) and Cell Quest software (BD Biosciences).

### Chemical treatment

H9 hPSCs were transfected with Control or PGRMC1 siRNA#1 as described above. After 24 h, cells were treated with 2 μM IWP-2 (Sigma-Aldrich) for 24 h as described previously^58^. Cells were harvested and analyzed on 72 h post-transfection.

### Statistical analysis

The paired samples t-test was a statistical procedure used to compare the difference between two populations, and a p-value of less than 0.05 was considered statistically significant. Data were expressed as the mean ± standard deviation (SD).

## Electronic supplementary material

Supplementary information
