# Supplementary material for: Progesterone Receptor Membrane Component 1 suppresses the p53 and Wnt/β-catenin pathways to promote human pluripotent stem cell self-renewal
Source: Sci Rep. 2018 Feb 14;8:3048. doi: 10.1038/s41598-018-21322-z (PMC5813096; doi:10.1038/s41598-018-21322-z)
Supplement: Supplementary file 1 — Supplementary information [file 41598_2018_21322_MOESM1_ESM.pdf]

Progesterone Receptor Membrane Component 1 suppresses the p53 and Wnt/β-catenin pathways to promote human pluripotent stem cell self-renewal

Ji Yea Kim[1]*, So Young Kim[1]*, Hong Seo Choi[1*], Min Kyu Kim[1], Hyun Min Lee[1], Young-Joo Jang[2], and Chun Jeih Ryu[1]

[1]Department of Integrative Bioscience and Biotechnology, Institute of Anticancer Medicine Development, Sejong University, Seoul, Korea.

[2]Department of Nanobiomedical Science, BK21 PLUS Global Research Center for Regenerative Medicine, Dankook University, Cheonan, Korea.

*These authors contributed equally to this work.

Correspondence and requests for materials should be addressed to CJR (email:cjryu@sejong.ac.kr) or to Y.-J.J (e-mail: yjjang@dankook.ac.kr).

Supplementary Figure 1

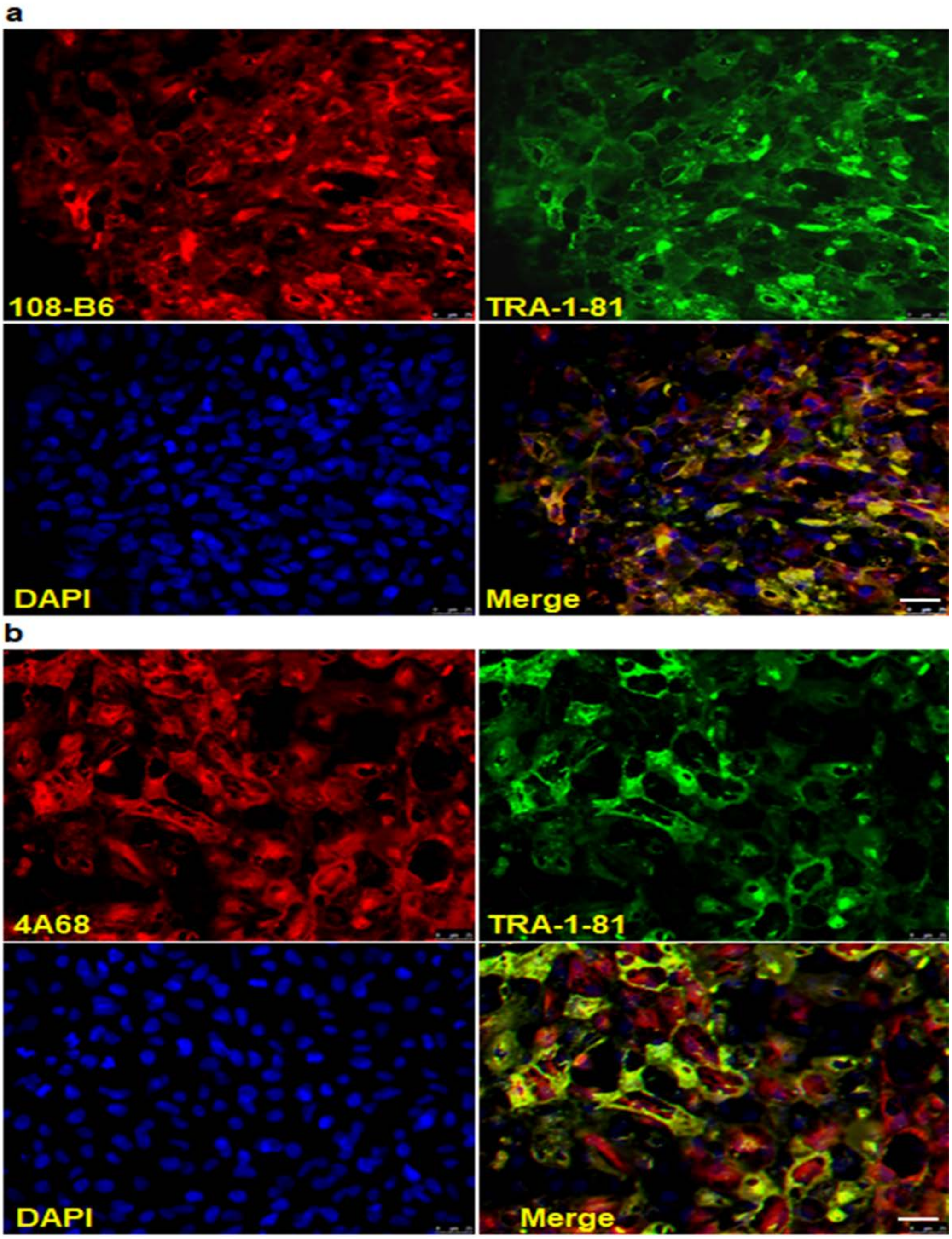

Supplementary Figure 1. Immunocytochemical analysis of hPSCs with 108-B6 (a) and 4A68 (b). H9 hPSCs were incubated with 108-B6 or 4A68 and TRA-1-81, a surface marker of undifferentiated hPSCs. The cells were then incubated with PE-conjugated anti-mouse IgG and FITC-conjugated anti-mouse IgM after fixation. Antibody staining is shown in green (108-B6 or 4A68) or red (TRA-1-81) and nuclear DAPI staining in blue. Scale bars are 25 μm.

Supplementary Figure 2

a

Match to: **gi|5729875** Score: **72** Expect: **0.017**

membrane-associated progesterone receptor component 1 [Homo sapiens]

Matching peptide shown in bold red

Start-End	Observed	Mr(expt)	Mr(calc)	Delta	Miss	Sequence
72-80	1104.6535	1103.6462	1103.5723	0.0739	1	R.DFTPAELRR.F
80-88	1089.6001	1088.5928	1088.5363	0.0565	1	R.RFDGVQDPR.I
89-102	1548.8593	1547.8520	1547.8745	-0.0225	1	R.ILMAINGKVFDVTK.G
105-119	1644.8389	1643.8316	1643.8096	0.0220	1	R.KFYGPEGPYGVFAGR.D
106-119	1516.7543	1515.7470	1515.7147	0.0323	0	K.FYGPEGPYGVFAGR.D

gi|5729875 (PGRMC1)

```
1    MAAEDVVATG ADPSDLESGG LLHEIFTSPL NLLLLGLCIF LLYKIVRGDQ
51   PAASGDSDDD EPPPLPRLKR RDFTPAELRR FDGVQDPRIL MAINGKVFDV
101  TKGRKFYGPE GPYGVFAGRD ASRGLATFCL DKEALKDEYD DLSDLTAAQQ
151  ETLSDWESQF TFKYHHVGKL LKEGEEPTVY SDEEEPKDES ARKND
```

b

Match to: **gi|5729875** Score: **89** Expect: **0.00033**

membrane-associated progesterone receptor component 1 [Homo sapiens]

Matching peptide shown in bold red

Start-End	Observed	Mr(expt)	Mr(calc)	Delta	Miss	Sequence
72-79	948.4390	947.4317	947.4712	-0.0395	0	R.DFTPAELR.R
72-80	1104.5470	1103.5397	1103.5723	-0.0326	1	R.DFTPAELRR.F
80-88	1089.5090	1088.5017	1088.5363	-0.0346	1	R.RFDGVQDPR.I
81-88	933.4140	932.4067	932.4352	-0.0285	0	R.FDGVQDPR.I
105-119	1644.7990	1643.7917	1643.8096	-0.0179	1	R.KFYGPEGPYGVFAGR.D
106-119	1516.7020	1515.6947	1515.7147	-0.0200	0	K.FYGPEGPYGVFAGR.D

gi|5729875 (PGRMC1)

```
1    MAAEDVVATG ADPSDLESGG LLHEIFTSPL NLLLLGLCIF LLYKIVRGDQ
51   PAASGDSDDD EPPPLPRLKR RDFTPAELRR FDGVQDPRIL MAINGKVFDV
101  TKGRKFYGPE GPYGVFAGRD ASRGLATFCL DKEALKDEYD DLSDLTAAQQ
151  ETLSDWESQF TFKYHHVGKL LKEGEEPTVY SDEEEPKDES ARKND
```

Supplementary Figure 2. Identification of 108-B6 and 4A68 antigens. NT-2/D1 cell lysates were immunoprecipitated with 108-B6 (a) or 4A68 (b), and were run on a 12% SDS-gel in non-reducing condition. The gels were then stained with Instant Blue staining solution. Approximate 28 kDa proteins were immunoprecipitated with 108-B6 or 4A68. The protein bands were excised, digested, and subjected to MALDI-TOF analysis. The search program MASCOT was used for protein identification by peptide mass fingerprinting. Tryptic peptides in red were matched to PGRMC1 protein.

Supplementary Figure 3

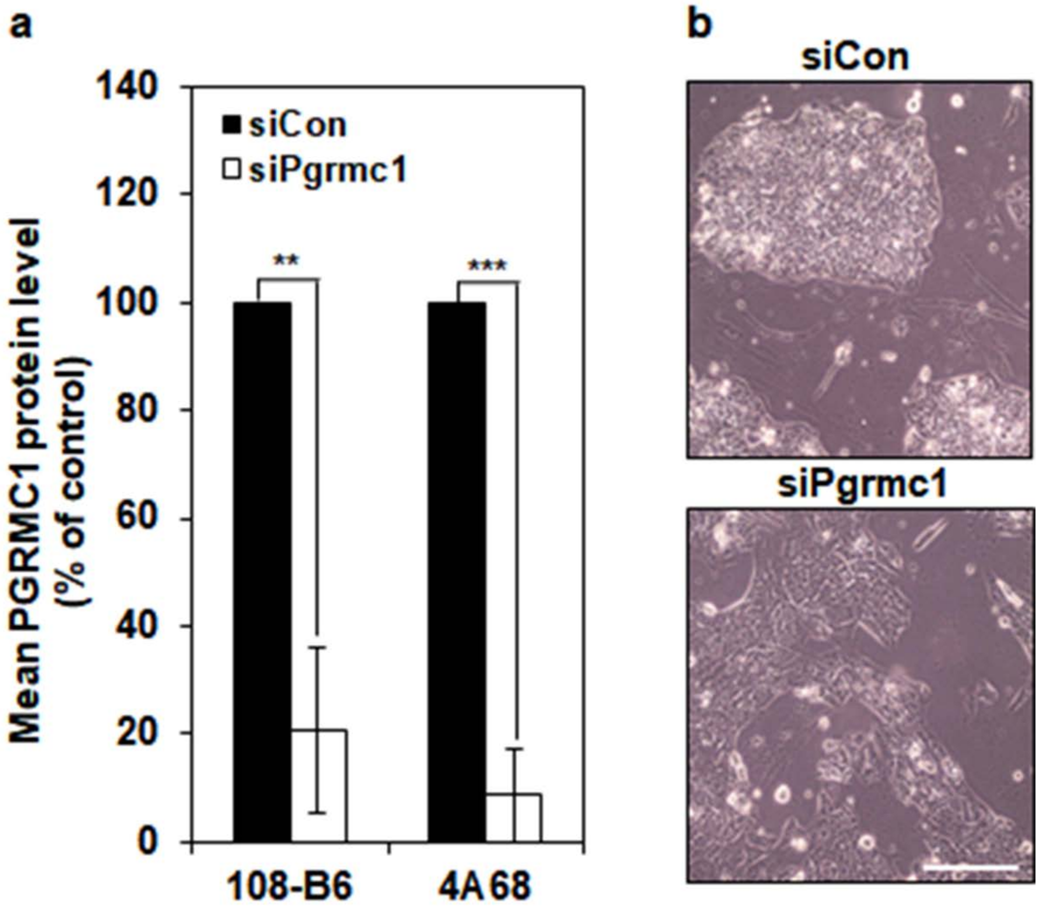

Supplementary Figure 3. Knockdown efficiency and cellular morphology of PGRMC1 knockdown hPSCs. (a) Quantitative analysis of efficiency of PGRMC1 knockdown in H9 hPSCs. H9 cells were transfected with either siCon or siPgrmc1 and subjected to Western blot analysis with 108-B6 and 4A68. The signal intensities were measured using the Image J software (n=3; ** p<0.01; *** p<0.005). (b) Cellular morphologies of control or PGRMC1 knockdown hPSCs. Scale bar is 200 μm.

Supplementary Figure 4

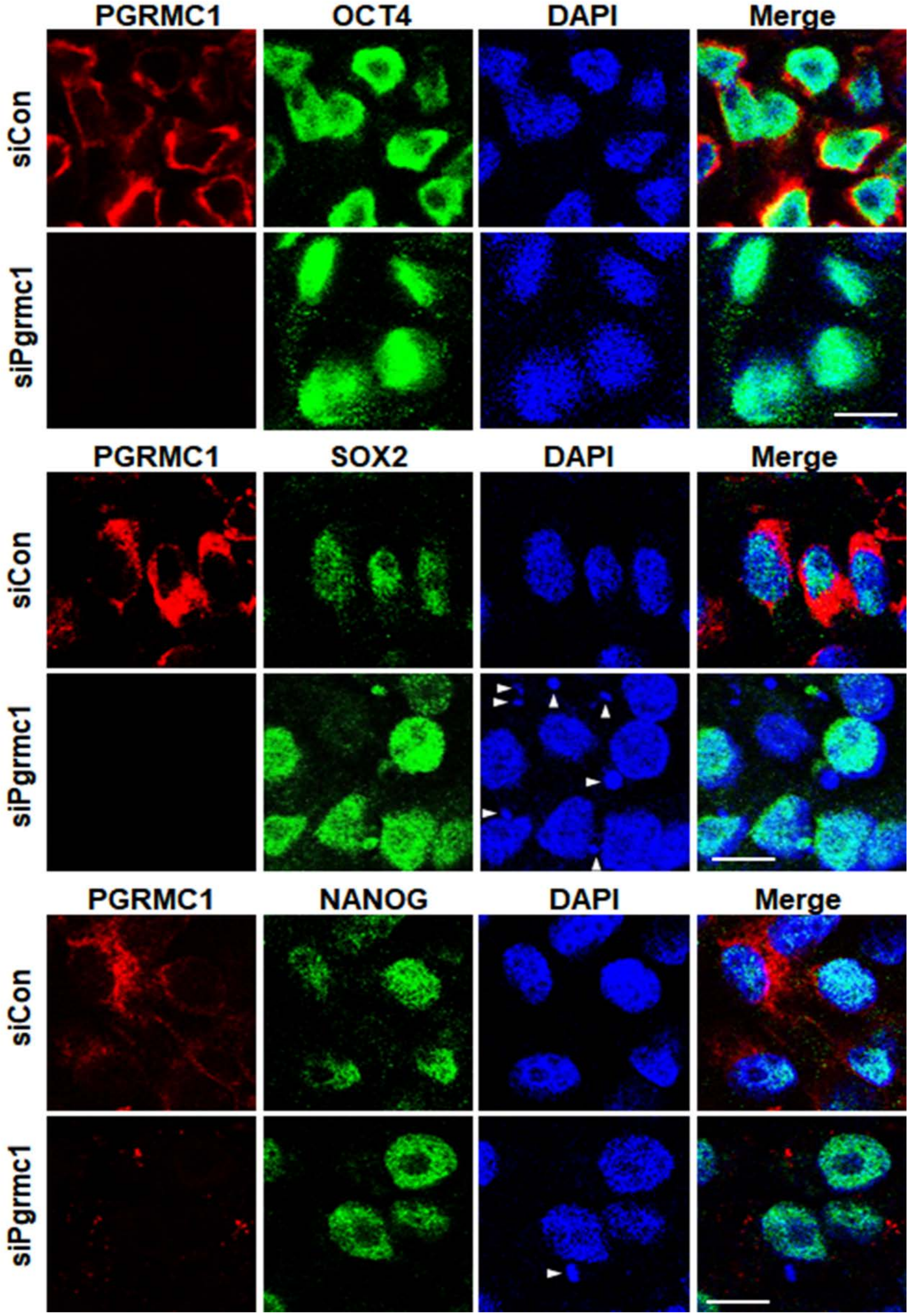

Supplementary Figure 4. Immunocytochemical analysis of PGRMC1, OCT4, SOX2, and NANOG in PGRMC1 knockdown hPSCs. Control or PGRMC1 knockdown hPSCs were fixed, permeabilized, and incubated with anti-OCT4, anti-SOX2, anti-NANOG, and anti-PGRMC1 (4A68). Cells were then incubated with Alexa 488-conjugated anti-rabbit IgG and Dylight 649-conjugated anti-mouse IgG. Nuclei were stained with DAPI. White arrowheads indicate abnormal micronuclei. Scale bars are 10 μm.

Supplementary Figure 5

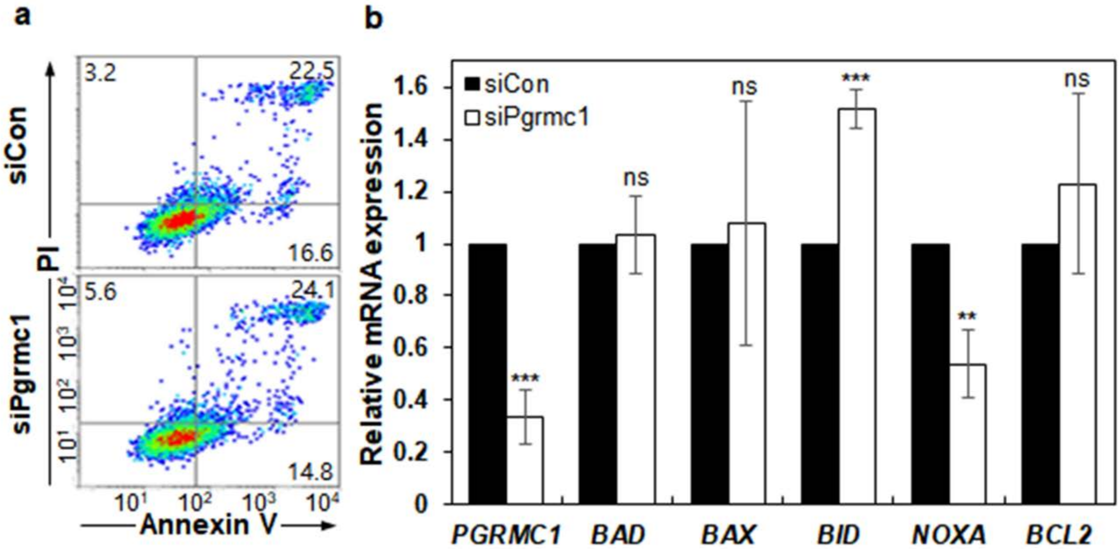

Supplementary Figure 5. Analysis of apoptosis of PGRMC1 knockdown hPSCs by flow cytometry and realtime PCR analysis. (a) Flow cytometric analysis of early and late apoptotic cells with annexin V and PI. Control and PGRMC1 knockdown hESCs were stained with PI and annexin V-FITC. (b) Real-time PCR analysis of mRNA levels of pro- and anti-apoptotic genes in PGRMC1 knockdown hPSCs. The graph represents the mean values of 5 independent experiments ± SD (n=5; *p<0.05; **p<0.01; *** p<0.005; ns, not significant).

Supplementary Figure 6

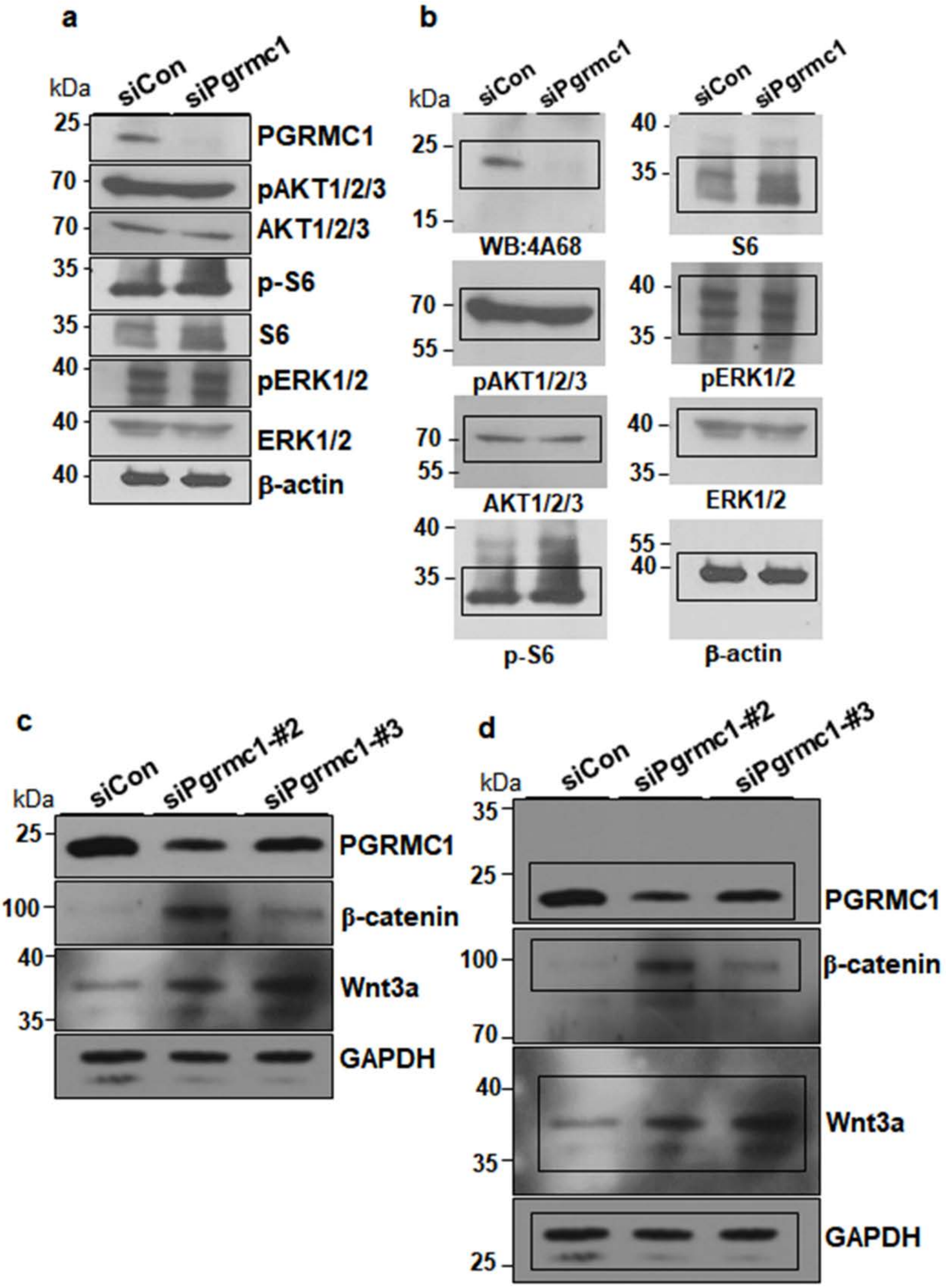

Supplementary Figure 6. Expression and phosphorylation analysis of PGRMC1, Akt1/2/3, S6, and ERK1/2 in control or PGRMC1 knockdown hPSCs and knockdown effects of PGRMC1 with different PGRMC1 siRNAs. (a) Expression and phosphorylation analysis of PGRMC1, Akt1/2/3, S6, and ERK1/2 in control or PGRMC1 knockdown hPSCs. Cell lysates were analyzed by Western blot analysis with indicated antibodies. β-actin was used as internal protein control and loading control. Full-length blots are presented in (b). Images are representative of at least three independent experiments. (c) Expression analysis of PGRMC1, β-catenin and Wnt3a in control or PGRMC1 knockdown hPSCs transfected with siRNA #2 and #3. Cell lysates were analyzed by Western blot analysis with indicated antibodies. DAPDH was used as internal protein control and loading control. Full-length blots are presented in (d). Images are representative of two independent experiments.

Supplementary Figure 7

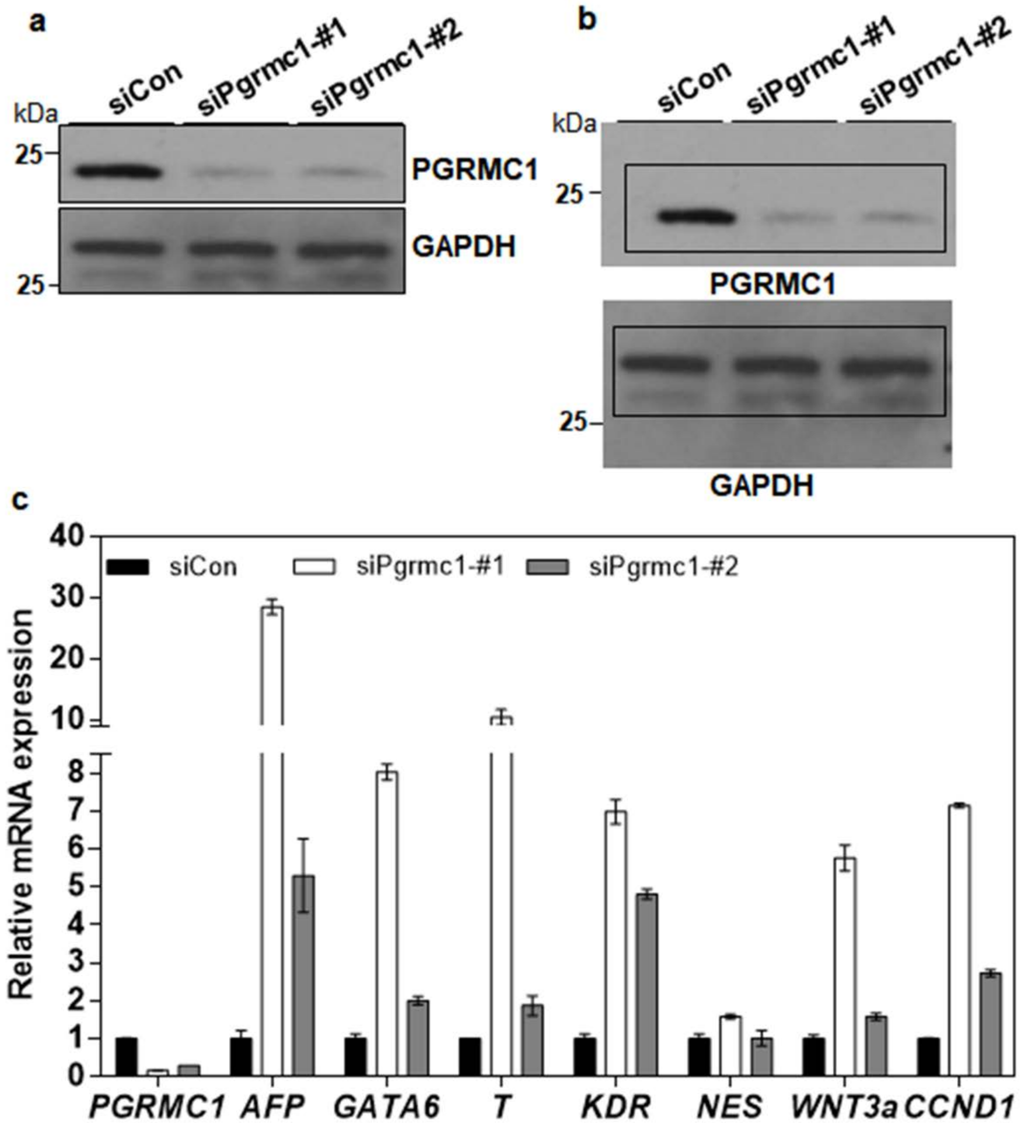

Supplementary Figure 7. PGRMC1 knockdown drives differentiation of CHA-hES4 hPSCs into multi-lineage cells. (a) PGRMC1 knockdown in CHA-hES4 hPSCs. CHA-hES4 cells were transfected with control (siCon) or PGRMC1 siRNAs #1 and #2 and subjected to Western blot analysis with anti-PGRMC1 antibody. DAPDH was used as internal protein control and loading control. Full-length blots are presented in (b). (c) Real-time PCR analysis of mRNA levels of early differentiation genes in control or PGRMC1 knockdown CHA-hES4 cells. The graph represents the mean values of two independent determinations ± SD.

Supplementary Figure 8

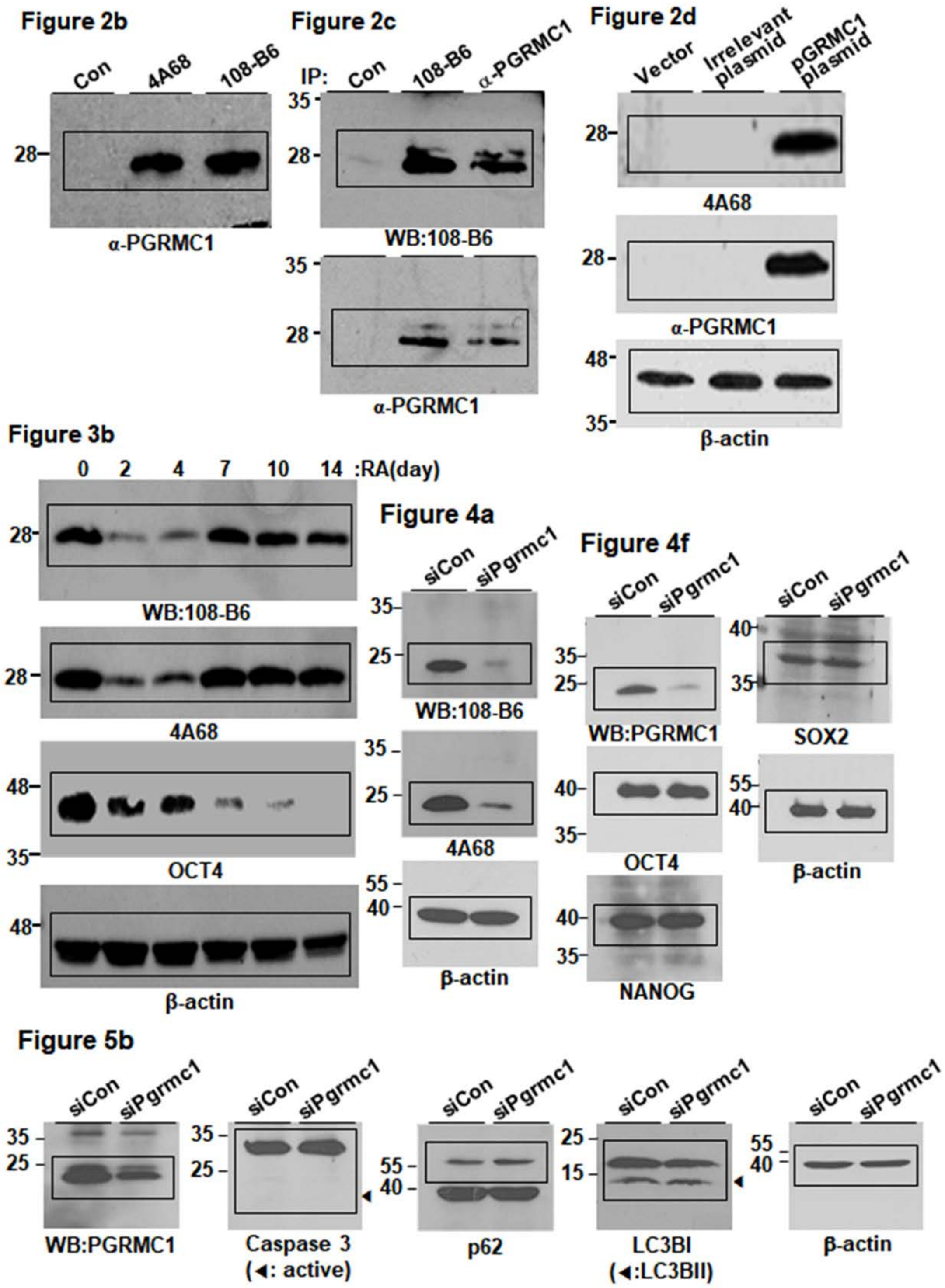

Supplementary Figure 8. Unprocessed scans of Western Blots of Figures 2b, 2c, 2d, 3b, 4a, 4f and 5b.

Supplementary Figure 9

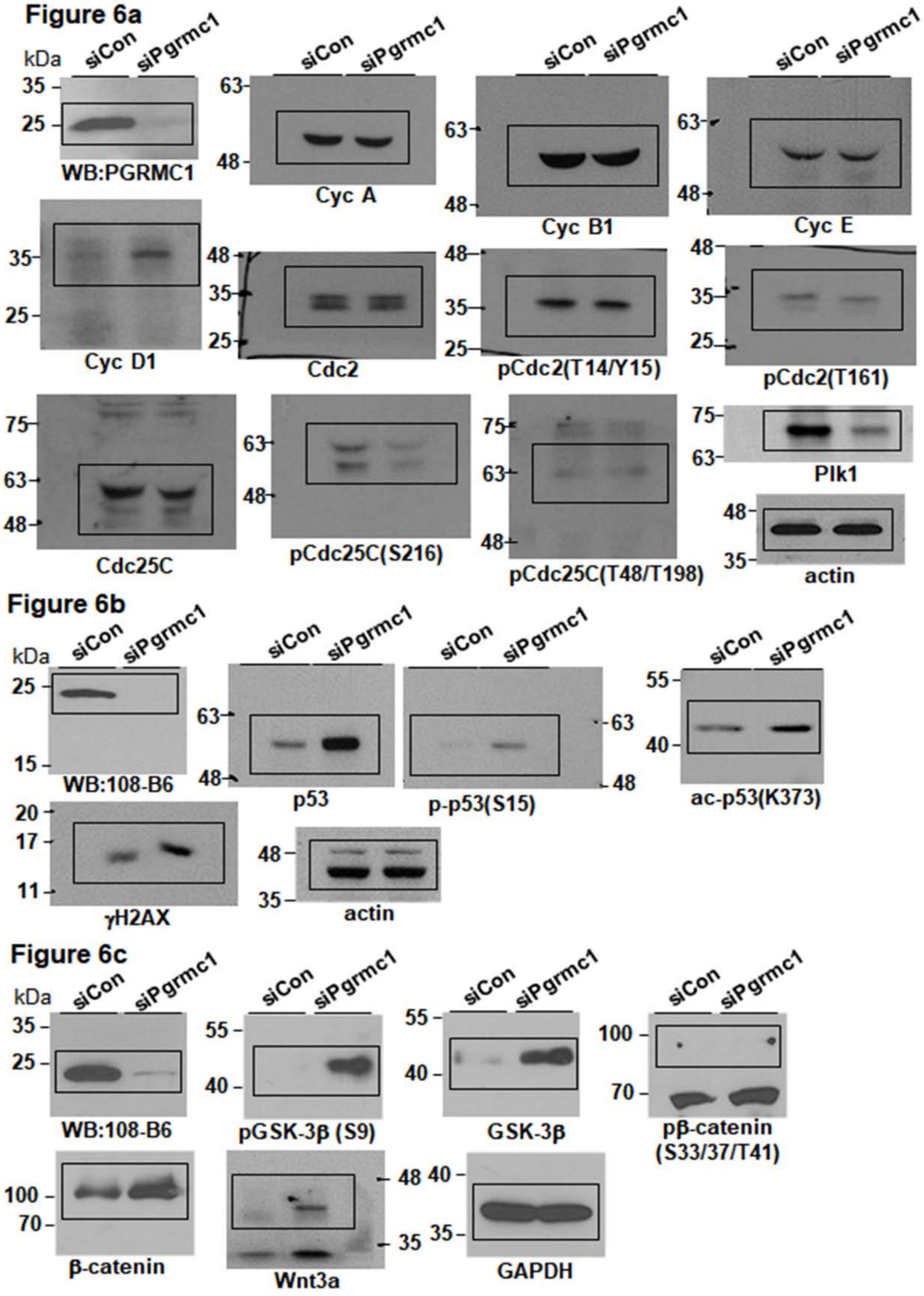

Supplementary Figure 9. Unprocessed scans of Western Blots of Figures 6a, 6b, and 6c.
